# Antiviral potential of crude extracts from two *Streptomyces* spp. against *Cucumber Mosaic Virus* infection under greenhouse conditions

**DOI:** 10.1038/s41598-025-31348-9

**Published:** 2025-12-23

**Authors:** Hadeel Osama, Mohamed E. El Awady, Radwan R. Khalil, Amro A. Farrag, Ahmed A. Hamed, Mohamed A. Nasr-Eldin

**Affiliations:** 1 Department of Botany and Microbiology, Faculty of Science, Benha University, Benha, 13511 Egypt; 2 Department of Microbial Biotechnology, National Research Centre, Giza, Egypt; 3 Virus and Phytoplasma Research Department, Plant Pathology Research Institute, Agricultural Research Center (ARC), Giza, 12619 Egypt; 4 Microbial Chemistry Department, National Research Centre, Dokki, Cairo, 12622 Egypt

**Keywords:** Marine actinomycetes, Ethyl acetate extracts, Control, CMV, Physiology, Pathogenesis-related genes, GC–MS, Microbiology, Physiology

## Abstract

Marine actinomycetes are a promising source for developing new antiviral agents for plant diseases, as they produce a wide variety of bioactive compounds. To date, antiviral activity of marine actinomycetes against plant viruses are rare. The present study proceeded to identify and characterize promising antiviral actinomycetes from Red Sea in Hurghada, Egypt, control *Cucumber Mosaic Virus* (CMV) and, profile the individual chemical components of bioactive crude extracts. Biological and molecular characterization was performed to identify CMV isolate using RT-PCR and coat protein (CP) gene nucleotide sequences analysis. In squash plants, antiviral, CMV optical density, biochemical responses, and resistance genes expression of *Streptomyces* extracts (SE1 and SE2) were performed against CMV by disease incidence, severity (%) assays, ELISA technique, physiological analysis, and real-time quantitative PCR (qPCR). Chemical profiling of the two *Streptomyces* extracts was investigated using GC–MS analysis. Antiviral activity was performed by curative (C), protective (P), and inactivation (I) techniques under greenhouse conditions. The obtained results of the morphological, biochemical, physiological and molecular level studies of the Streptomycetes isolates ph6 and MARH showed similarity towards the species of *Streptomyces* and identified as *Streptomyces variabilis* strain ph6 (OQ283766) and *Streptomyces* sp. strain MARH (OQ283775). Protective treatments (P: SE1 and P: SE2) resulted in disease suppression of 100%, while curative treatments (C: SE1 and C: SE2) by 87% and 100%, and inactivation treatments (I: SE1 and I: SE2) by approximately 70%, and 80% respectively compared to mock-inoculated plants. The absorbance values of ELISA at 405 nm for P: SE1 and P: SE2 were 0.191 ± 0.02, and 0.187 ± 0.00 respectively compared to CMV-infected plants which recorded 0.854 ± 0.00. Levels of ascorbate peroxidase (APX), catalase (CAT) enzymes, proline content, total photosynthetic pigments, and total phenolic compounds were significantly increased in P: SE1 and P: SE2 treated squash plants compared to CMV-infected plants. Moreover, P: SE1 and P: SE2 increased membrane stabilization and reduced electrolyte leakage. The pathogenesis-related (PR) gene such as PR-b1 was overexpressed by about 3.09-, and 10.37-fold increases and PR-2 by 1.07-, and 1.66-fold increases for P: SE1 and P: SE2 respectively compared to the CMV-infected control group. Chemical profiling of the ethyl acetate extracts of *Streptomyces variabilis* and *Streptomyces* sp. MARH authenticated the presence of constituents such as 1,3¬Dinitro¬2-imidazolidinone (42.60%), Nephthoside-1,2’,3’,4’-Tetraacetate (76.18%), Tetraphenylporphyrinato dichlorotitanium(IV) (12.42%) and L-Lysine (17.01%) respectively. Our results showed that *Streptomyces variabilis* and *Streptomyces* sp. MARH are promising strains for production of antiviral natural products, increasing the phenolic compounds, activity of the antioxidant enzymes, and induction the expression of pathogenesis-related genes to generate systemic acquired resistance (SAR) in squash plants. They also supported the potential use of their extracts as an environmentally friendly novel bio-virocides to sustainably stop the spread of plant viruses.

**Supplementary Information:**

The online version contains supplementary material available at 10.1038/s41598-025-31348-9.

## Introduction

Plant virus disease represents a significant agricultural concern and poses a substantial risk to the growth and development of plants. *Cucumber mosaic virus* (CMV) is the type member of the genus Cucumovirus in the family Bromoviridae. The genome of CMV is made up of three single-stranded, positive-sense RNAs, encoding five proteins with an icosahedral particle size of 30 nm in diameter^1^. CMV has a worldwide distribution and a host range of over 1200 plant species, including various squash species (*Cucurbita pepo* and *Cucurbita moschata*) and cucurbits species like cucumbers (*Cucumis sativus*), melons (*Cucumis melo*), and watermelons (*Citrullus lanatus*). Additionally, the virus is transmitted by more than 60 aphids and infected seeds^2^. CMV causes a variety of symptoms that differ based on the viral strain and plant species, but frequently comprise leaf mosaic, yellowing, mottling, vein yellowing, and growth stunting because of its wide host range, quick evolution, and disastrous effects on crop and fruit quality. It is considered one of the most aggressive and destructive RNA viruses affecting cucurbit crops worldwide. Mediterranean countries like Egypt, which is ranked among the top ten producers of cucurbit crops, are severely affected by this virus^3^.

Currently, there is no direct cure available on the market for viral infections in plants, and there are still several obstacles in the way of creating extremely effective antiviral agents^4^. Moreover, the excessive application of synthetic agro-pesticides to control the virus and its vectors has resulted in numerous harmful consequences, such as environmental pollution, adverse effects on human health, and the development of resistance in the pathogen. Altogether, sustainable agriculture is exploring new biocontrol agents and cost-effective methods for implementing these agents or their natural bioactive metabolites in strategies for disease management.

Recently, *Streptomyces* has emerged as a compelling potential agent and environmentally friendly option for sustainable agriculture, serving as a promising candidate for the biocontrol of various phytopathogens^4^. *Streptomyces* species are well recognized as promising biological agents for managing plant diseases offering an alternative to chemical pesticides^5^. The primary biological characteristic of *Streptomyces* is the production of various bioactive secondary metabolites, including antibacterial, herbicidal, antifungal, and anticancer substances and plant growth promoters such as auxin, cytokinin and gibberellin^6^. These secondary metabolites-producing bacteria can be applied as biofertilizers in biocontrol management strategies against plant virus diseases. Several species of *Streptomyces* have shown their efficiency against RNA viruses. Some bioactive compounds (Ningnamycin, Glycoprotein GP-1, and ε-Poly-L-lysine) derived from *Streptomyces* strains that isolated from soil samples have antiviral activities against novel plant viruses^7–9^.

Virus infection causes physical, physiological, and biochemical changes in host cells, producing several symptoms (mosaics, necrosis, and deformities of seeds, leaves, and crops). Furthermore, the virus induces physiological alterations in plant cellular processes, such as the manipulation of chloroplast proteins, hormones, and the accumulation of reactive oxygen species (ROS)^10,11^. A protective enzyme system that includes mainly (ROS)-eliminating enzymes, such as catalase (CAT), and ascorbate peroxidase (APX) induce plant resistance to infection^12^. Pathogenesis-related proteins (PRs) represent a category of substances in plants that have the potential to combat diseases. These PRs can be synthesized or accumulated by plants in response to pathogen infection or when treated with certain compounds, thereby demonstrating resistance to infections^13^. Salicylic acid (SA) controls the overexpression of many pathogenesis-related (PR) proteins in plants as well as their systemic acquired resistance (SAR), which includes antioxidant enzyme accumulation and cell-wall fortification^14–16^.

*Streptomyces* spp. metabolites have been shown to protect plants from bacterial and fungal diseases. Research is still being conducted on the effectiveness against viruses^17^. Many reports have shown that *Streptomyces* species have few uses in bio-controlling plant viruses, and it is still unclear how they might work as antiviral agents^18^. Because of their specific metabolic pathways brought about by the unique marine environment, these species are a significant source for the discovery of new natural compounds for biotechnology and agriculture. This is the first report about the antiviral activity of crude extracts derived from *Streptomyces* spp. isolated from marine environment against CMV.

Therefore, the present research aimed to isolate and characterize marine *Streptomyces* spp. from the Red Sea of Egypt and explore their antiviral potential against CMV in squash plants by using three antiviral methods under greenhouse conditions. In addition, this study has attempted to clarify the defense mechanisms that are triggered in crude extracts-treated plants, including the change in antioxidant enzyme activities and biochemical indicators associated with defense. Expression of pathogenesis-related genes such as PR-b1 and PR-2 was also investigated. Furthermore, the bioactive compounds of their ethyl acetate extracts were identified using gas chromatography–mass spectrometry (GC–MS) analysis.

## Materials and methods

### Sampling and isolation of Streptomycetes

Samples were obtained from the silt located at a depth of 1 cm along the shore of the Red Sea in Hurghada, Egypt (27°18′22.02" N, 33°43′44.8" E). Samples were diluted serially following the protocol described by Hayakawa & Nonomura^19^. The diluted sample was then plated onto a starch nitrate agar medium, which consisted of the following components in g/l: starch (10), K_2_HPO_4_ (1.0), MgSO_4_.7H_2_O (0.5), NaCl (0.5), KNO_3_ (2.0), CaCO_3_ (2.0), FeSO_4_.7H_2_O (0.01), and agar (20.0). These components were dissolved in 750 mL of seawater and then brought to a final volume of 1 L with 250 mL of distilled water. The pH of the medium was adjusted to 7^20^. The selection of colonies on agar plates was based on their appearance and their potential for secondary metabolite production. Additionally, the isolates of *Actinomycetes* were inoculated onto a medium consisting of starch nitrate agar and subjected to incubation at a temperature of 30 °C for a duration of 72 h. The *Streptomyces* isolates were chosen and collected based on the morphological characteristics exhibited by their colonies. Streptomycetes have special colonies, which are rounded, convex colonies with rooting growth into media covered with powdery spores.

### Production of crude extracts from Streptomyces

The *Streptomyces* strains were cultivated on ISP2 agar plates at a temperature of 28 °C for a duration of 3 days in a scale-up fermentation study aimed at generating secondary metabolites. The rice was sterilized using an autoclave and then inoculated with a sample from a well-developed agar subculture of the *Streptomyces* strains. This process was carried out in a 1-L Erlenmeyer flask. Prior to being collected, the flasks were maintained at a temperature of 28 °C for a duration of 15 days. The crude extract was obtained by extracting secondary metabolites from a rice medium using ethyl acetate, followed by filtering and evaporation utilizing a rotary evaporator^21^.

### Identification of Streptomycete isolates

The half-leaf method was used to evaluate the crude extracts’ antiviral activity against CMV infection on *Chenopodium amaranticolor* plants, which were obtained from the Department of Virus and Phytoplasma Research, Agricultural Research Center, Giza, Egypt, and helped determine the potential isolates (ph6 and MARH)^22^.

For a half-leaf assay, the age of 2–4 leaf stage was used, with 300 uL from CMV inoculum (1g infected leaf/10 ml 10 mM sodium phosphate buffer (pH 7.2)), using a gentle mechanical rub with an abrasive (Carborundum powder (600 mesh)) to introduce the inoculum, involving 3 leaves/plants for each treatment, and then counting the resulting local lesions after 7 days of inoculation. The isolates with the strongest antiviral activity were identified by their morphological, physiological, and biochemical characteristics^23–25^ and confirmed by the 16S ribosomal RNA gene technique (16S rRNA) and were chosen for further experiments. Firstly, genomic DNA was extracted from log-phase grown strains (ph6 and MARH), and the 16S rRNA gene was amplified using the polymerase chain reaction (PCR) technique. The primers used for amplification are listed in Table 1**.** The sequencing procedure was conducted with the BigDye terminator cycle sequencing kit (Applied Biosystems, USA). The PCR condition was as follows: 4 min at 96 °C, followed by 30 cycles of 30 s at 94 °C, 30 s at 57 °C and 1 min at 72 °C, and a final extension step at 72 °C for 10 min. used for amplification are listed in Table 1. The sequencing procedure was conducted with the BigDye terminator cycle sequencing kit (Applied BioSystems, USA). The sequencing products were separated using an Applied Biosystems model 3730XL automated DNA sequencing device, manufactured by Applied Biosystems in the United States. The obtained 16 s rRNA sequence was deposited into the GenBank database and subsequently subjected to comparison with other 16 s rRNA sequences available in the National Centre for Biotechnology Information (NCBI) (https://www.ncbi.nlm.nih.gov/) using the BLAST program. The selection and alignment of bacterial strains with the highest similarity to the 16S rRNA gene of our isolates were performed to construct an appropriate phylogenetic tree. The nucleotide sequence of the 16 s rRNA gene from our isolates has been submitted to the GenBank database, NCBI, under the accession numbers OQ283766 and OQ283775.

**Table 1:** The primers used in this study.

No	Name	Direction	Sequence (5’- > 3’)	Length	Reference
1	16S ribosomal RNA	Forward primer	GAGTTTGATCCTGGCTCAG	19	^92^
		Reverse primer	GGTTACCTTGTTACGACTT	19	
2	Cucumber mosaic virus-coat protein	Forward primer	TTGAGTCGAGTCATGGACAAATC	23	^93^
		Reverse primer	AACACGGAATCAGACTGGGAG	21	
3	Elongation factor 1-alpha	Forward primer	ATTCGAGAAGGAAGCTGCTG	20	^42^
		Reverse primer	TTGGTGGTCTAAACTTCCAC	20	
4	Pathogenesis-related protein PR-b1	Forward primer	AACTCTGGCGGACCTTAC	18	^94^
		Reverse primer	GACTTCCTCCACACTACT	18	
5	Pathogenesis-related protein PR-2	Forward primer	TCTTGGTCTTCTTGTGCC	18	^95^
		Reverse primer	GAGCATCAAGTGAACCTC	18	

### Source of the viral isolate

The CMV isolate utilized in this investigation which had previously been isolated from diseased cucumber plants, was kindly provided by the Department of Virus and Phytoplasma Research, Plant Pathology Research Institute, Agricultural Research Center, Giza, Egypt. The CMV inoculum was produced by extracting infectious sap from infected plant leaves and treating it with 10 mM sodium phosphate buffer (pH 7.2) and 0.1% sodium sulfite^26^.

### Molecular detection of CMV

#### Reverse transcription-polymerase chain reaction (RT-PCR)

Total RNA was extracted from the tested squash leaf samples using the Total RNA Mini Kit plant (Geneaid) following the manufacturer’s instructions and stored at −20 °C until testing. To detect CMV, primers were used to amplify 678 bp of its coat protein gene **(**Table 1**)**.

One-step RT-PCR was carried out by using the Verso TM one step RT-PCR kit (Thermo Scientific) in a 25 µL reaction mixture that contained 3 µL of RNA sample, 12.5 µL of one step PCR master mix (2x), 2 µL of 10 µM of each primer, 0.25 µL of Verso enzyme mix, 2.5 µL of RT Enhancer, and 2.75 µL of nuclease-free water.

This was the state of the reaction conditions: A 15-min cDNA synthesis stage is performed at 50 °C, followed by denaturation at 95 °C for two minutes. Thirty cycles of 94 °C, 50 °C, and 72 °C are then performed, with the final elongation step taking place at 72 °C for ten minutes. PCR products were analyzed by electrophoresis in a 1.0% agarose gel stained with ethidium bromide using a 100 bp DNA ladder (BIOMATIK).

### Gel clean and nucleotide sequence analysis

RT-PCR products of CMV isolate were cut from the gel and purified using QIAGEN gel extraction kit according to the manufacturer’s guidelines, purified samples were sent to Macrogen Company, Korea for sequencing. Nucleotide sequences were analyzed and aligned using DNAMAN 7 program with other CMV sequences available on the GenBank database and phylogenetic tree was constructed.

### Reactions of different plant species to CMV

Different plant species from two families were mechanically inoculated with CMV-infectious crude sap expressed from squash plants in an insect-proof greenhouse. The infection of the test plants was identified at 7-, 14-, and 21-days following virus inoculation by monitoring the occurrence of local and systemic symptoms. The Double-antibody sandwich enzyme-linked immunosorbent assay (DAS-ELISA) method was used to confirm the CMV infection.

### Greenhouse experimental design

Virus-free seeds of squash (*Cucurbita pepo* L.) plants, cultivar New Eskandrany H1, were obtained from the Virology Lab at the Agriculture Research Center (ARC), Egypt. The squash seeds were planted in plastic pots (30 cm in diameter) containing 4 kg of clay soil and maintained in an insect-proof greenhouse condition. After 14 days, the true upper leaves of each squash plant were mechanically inoculated with the CMV^26^. The extracted metabolites form *Streptomyces variabilis* strain ph6 and *Streptomyces* sp. strain MARH were dissolved in 5% Dimethyl sulfoxide (DMSO) at concentration 50 mg/mL while the 5% DMSO was used as negative control. Each plant was sprayed with 3 ml. Five groups were formed for the experiment, with three replicates in each group **(**Table 2**)**. The first group (control treatment group) consisted of mock plants. The second group (CMV-infected group) consisted of squash plants that had been mechanically infected with the CMV. Plants inoculated with CMV were treated independently by foliar spraying crude extracts of strain ph6 (C: SE1), strain MARH (C: SE2), and a 5% DMSO solution only (C: DMSO) 24 h after CMV infection, as the third group, which was the curative treatment group. The fourth group (protective treatment group) was separately sprayed with crude extracts of strain ph6 (P: SE1), strain MARH (P: SE2), and 5% DMSO solution only (P: DMSO) 24 h before CMV inoculation. The fifth group (inactivation treatment group) was treated by rubbing the mixture of CMV + crude extracts of strain ph6 (I: SE1), CMV + crude extracts of strain MARH (I: SE2), and CMV + 5% DMSO solution only (I: DMSO) (1:1 v/v) after 15 min of mixing. Every day, all squash plants were monitored, and the progression of the viral disease symptoms was recorded. Every experiment was conducted twice.

**Table 2:** Greenhouse experiment scheme.

Group number	Group name	Treatment*	
1	Negative control	Mock	Squash plants were foliar sprayed with 5% DMSO solution only and mocked with viral inoculation buffer (Non inoculated plants)
2	Positive control	CMV-infected	Squash plants were inoculated with CMV (Inoculated plants)
3	Curative	C: SE1	Squash plants were foliar sprayed with crude extract from *Streptomyces variabilis* 24 h after CMV inoculation
		C: SE2	Squash plants were foliar sprayed with crude extract from *Streptomyces* sp. MARH 24 h after CMV inoculation
		C: DMSO	Squash plants were foliar sprayed with 5% DMSO solution 24 h after CMV inoculation
4	Protective	P: SE1	Squash plants were foliar sprayed with crude extract from *Streptomyces variabilis* 24 h before CMV inoculation
		P: SE2	Squash plants were foliar sprayed with crude extract of *Streptomyces* sp. MARH 24 h before CMV inoculation
		P: DMSO	Squash plants were foliar sprayed with 5% DMSO solution 24 h before CMV inoculation
5	Inactivation	I: SE1	Squash plant leaves were rubbed with the mixture of CMV + crude extract from *Streptomyces variabilis* after 15 min of mixing
		I: SE2	Squash plant leaves were rubbed with the mixture of CMV + crude extract from *Streptomyces* sp. MARH after 15 min of mixing
		I: DMSO	Squash plant leaves were rubbed with the mixture of CMV + 5% DMSO solution after 15 min of mixing

* (C) Curative, (P) Protective and (I) inactivation treatments; (SE1) and (SE2) bioactive crude extracts from *Streptomyces variabilis* and *Streptomyces* sp. MARH respectively.

### Disease incidence and severity (%)

The disease incidence of squash (*Cucurbita pepo* L.) plants was observed and recorded for control, treated and CMV-inoculated plants every day until 30 days post virus inoculation (DPVI).

1 $$Disease\,incidence\,DI(\% ) = \frac{Number\,of\,\inf ected\,plants}{{Number\,of\,total\,evaluated\,plants}} \times 100$$

The severity of symptoms was assessed 21 DPVI based on a scale proposed by Ryu et al.^27^, of 0–10 as follows: 0 = no symptoms, 2 = Vein clearing, 4 = Mild mosaic, 6 = severe mosaic, 8 = severe mosaic and chlorosis 10 = severe mosaic, chlorosis and malformation.

Disease severity (DS) values were calculated using the following formula according to Yang et al.^28^.

2 $$Disease\,severity\,DS(\% ) = \frac{\sum Disease\,grade\,X\,Number\,of\,plants\,in\,each\,grade\,}{{(Total\,number\,of\,\,plants\,X\,Higest\,disease\,grade)}} \times 100$$

### Quantitative detection of CMV concentration by DAS-ELISA

Double-antibody sandwich enzyme-linked immunosorbent assay (DAS-ELISA) was utilized to detect the CMV infection and to evaluate the virus concentration in squash leaves after 21 DAVI^29^. ELISA was used to examine newly emerged leaves from both inoculated and non-inoculated plants (five replicates per treatment). The ELISA reading value for the CMV was 0.854 OD, compared to 0.181 OD for negative control plants.

### Metabolic indicators for squash resistance

#### Determination of photosynthetic pigments

Fresh squash leaves were used to extract the photosynthetic pigments chlorophyll a, chlorophyll b, and carotenoids, which were then estimated colorimetrically in accordance with Metzner et al.^30^. The amount of pigment contained in the leaves was determined as μg/g dry weight (μg/g D.wt.).

### Determination of antioxidant enzymes (APX and CAT)

#### Enzyme extraction

Samples were prepared as described by Mukherjee & Choudhuri^31^. 2.5 g of fresh squash leaves were finely ground using liquid nitrogen by pestle in a chilled mortar; the frozen powder was mixed with 10 mL of 100 mM phosphate buffer, pH 7.0, comprising 0.1 mM Na_2_EDTA and 0.1 g of PVP. The homogenate was filtered then centrifuged at 18,000 xg for 10 min. The supernatant was taken and stored at 4 °C for analysis of catalase (CAT) and ascorbate peroxidase (APX) activities.

- a) Determination of catalase (CAT) activity

Catalase activity (CAT; EC 1.11.1.6) was analyzed using Aebi method^32^,. The reaction mixture (3 mL) contained 500 µL of enzyme extract, 30% (w/v) H_2_O_2_, and phosphate buffer (50 mM, pH 7.0). CAT activity was measured using a UV spectrophotometer set to 240 nm to measure the rate change of H_2_O_2_ absorbance in 1 min. CAT activity was defined as μM H_2_O_2_ oxidized g^−1^ fresh weight min^−1^^33^.

- b) Determination of ascorbate peroxidase (APX) activity

Ascorbate peroxidase activity (APX; EC 1.11.1.11) was analyzed by estimating the reduction in absorbance at 290 nm for 60 s of ascorbic as ascorbic acid oxidized. APX activity was defined as mM ascorbate oxidized g^−1^ fresh weight min^−1^^34^.

### Estimation of total phenol

Total phenols were analyzed using the Folin–Ciocalteu reagent^35^. Exactly 0.5–1.0 g of dried squash leaves was ground with ten times the volume of 80% ethanol. The homogenate was centrifuged for 20 min at 12.000 xg, and the supernatant was separated. After centrifuging and saving the supernatant, the residue was extracted again using a 5-times volume of 80% ethanol. After that, the residue was dissolved in 5 mL of distilled water, and the supernatant was dried out by evaporation. Test tubes were pipetted with various aliquots (0.2–2 mL). Each tube’s volume was brought to 3 mL using water. Add 500 µL of Folin–Ciocalteu reagent. After 3 min, each tube was filled with 2 mL of a 20% NaCO₃ solution and thoroughly mixed. After a minute of incubation in boiling water, the tubes were cooled. A spectrophotometer was used to read the absorbance at 650 nm.

### Determination of proline content

The proline content was examined using the methodology of Bates et al.^36^. After homogenizing one gram of fresh, macerated squash leaves in ten milliliters of 3% aqueous sulfosalicylic acid, the mixture was filtered through filter paper. After combining the filtrates (2 mL) with 2 mL of glacial acetic acid, 2 mL of freshly made acid-ninhydrin reagent was added. The test tubes were incubated for one hour at 100 °C in a water bath. After adding 4 mL of toluene to the reaction mixture, it was vortexed for 15 to 20 s. The toluene-containing chromophore was heated to room temperature after being aspirated from the aqueous phase. Toluene was used as a blank, and a spectrophotometer was used to measure the absorbance at 520 nm.

### Evaluation of membrane stability and electrolyte leakage

#### Electrolyte leakage (EL)

The total inorganic ions leaked out from the squash leaves were assessed according to the Srivastava & Srivastava^37^ method. In a boiling tube with 10 mL of deionized water, 20 leaf discs were added. The tubes were placed in a water bath and incubated for 30 min at 45 °C (ECa) and 55 °C (ECb). The EC values were then determined using a conductivity meter. Following a 10-min boil at 100 °C, the contents’ EC was once more measured as ECc. Using the following formula, the electrolyte leakage was determined:

3 $$Electrolyte\,leakage\,(\% ) = \frac{ECb - ECa}{{ECc}} \times 100$$

### Membrane stability Index (MSI)

Two sets of 200 mg squash leaves were added to 10 mL of double-distilled water for MSI analysis. A conductivity meter was used to measure the electrical conductivity C_1_ of one set after it had been heated for 30 min at 40 °C in a water bath. On the other hand, the conductivity of the second set was also measured after it was incubated for 10 min at 100 °C in a water bath (C_2_). The formula presented by Sairam^38^ was used to calculate MSI:

4 $$MSI = \left[ {1 - (\frac{c1}{{c2}}) \times 100} \right]$$

### Detection of pathogenesis-related (PR) genes expression under CMV challenge using real-time quantitative PCR (qPCR)

To investigate the expression of the PR-b1 and PR-2 genes in squash plants cv. New Eskandrany H1 at 21 DPVI, total RNA was extracted using fresh leaf samples that were specifically collected for each treatment, in accordance with the supplier’s instructions (Norgen Biotek Corp Total RNA Purification Kit, Canada). Approximately 1 µg of total RNA (pretreated with DNase I) was reverse transcribed into single-stranded cDNA. Total volume was adjusted to 20 µL using the above cDNA as templates. The components of the reaction mixture utilized in the current study to determine the expression of different genes are as follows: 10 μL Maxima SYBR Green/ROX qPCR Master Mix (Thermo Scientific™), 1 µL forward primer, 1 µL reverse primer, 2 μL cDNA template, and 6 μL nuclease-free water. In accordance with Suzuki et al.^39^, real-time PCR amplifications were carried out using the BIO-RAD CFX Connect Real-Time PCR Detection System, Singapore, to detect the expression of PR-b1^40^ and PR-2^41^ as follows: 10 min at 95 °C, 40 cycles of (15 s at 95 °C, 60 s at 60 °C, 60 s at 72 °C). The fluorescence data were collected throughout the 72 °C extension, and a melt curve analysis was used to confirm the specificity and identity of the RT-PCR products: An additional melting step, or dissociation stage, was introduced. It ramps from 72 to 99 °C, increasing by 1 °C per step, and requires waiting periods of 30 s for the first step and 5 s for each subsequent step. Relative gene expression was quantified using the standard curve method, and the transcript accumulation of each gene was normalized to the housekeeping gene Elongation factor 1-alpha^42^. The gene-specific primers used in this experiment are listed in Table 1. The results were the mean of three replicates/treatments. The normalized ΔCT data were utilized to calculate the relative gene expression fold change by applying a selected calibrator (reference sample). Using the 2^-∆∆Ct^ algorithm, the relative expression ratio was precisely measured and quantified^43^.

### Identification of bioactive compounds of Streptomyces spp. crude extracts through Gas Chromatography–Mass Spectrometry (GC–MS)

The ethyl acetate extracts from *Streptomyces* spp. were analyzed using a GC–MS which was carried out using a Thermo Scientific, Trace GC Ultra/ISQ Single Quadrupole MS, TG-5MS fused silica capillary column (30 m × 0.251mm × 0.1 mm film thickness). For GC/MS detection, helium gas was used as the carrier gas in an electron ionization system with an ionization energy of 70 eV at a steady flow rate of 1 mL/min. The injector and MS transfer line temperature was set to 280 °C. The oven was programmed to reach and maintain 45 °C for two minutes. The temperature was then set to rise at a rate of 7 °C per min to 150 °C, 5 °C per minute to 270 °C (held for 2 min), and 3.5°C per minute to 310 °C, the final temperature (held for 10 min). The quantification of each identified component was examined using a percent relative peak area. A preliminary identification of the compounds was performed by comparing their mass spectra and relative retention times with the NIST and Wiley library data of the GC/MS system.

### Statistical analysis

Data from all experiments were statistically analyzed using the MSTAT-C Statistical Software Package. Analyses were obtained using two-way ANOVA, and the experimental design was completely randomized. Where the F-test showed significant differences among means, Duncan’s multiple range test^44^ at the p ≤ 0.05 level was used to verify the significance of means for all traits recorded.

## Results

### Screening of Streptomycete

*Streptomycetes* represent the predominant bacterial species employed in the process of fermenting bioactive compounds. A total of seven isolates were obtained from sand samples collected from the Red Sea in Hurghada, Egypt. According to chlorotic local lesion inhibitions on the inoculated leaves of *Chenopodium amaranticolor*, only two isolates, ph6 and MARH, could show high activity for controlling CMV infection.

### Identification of promising Streptomyces isolates

**Fig. 1:**
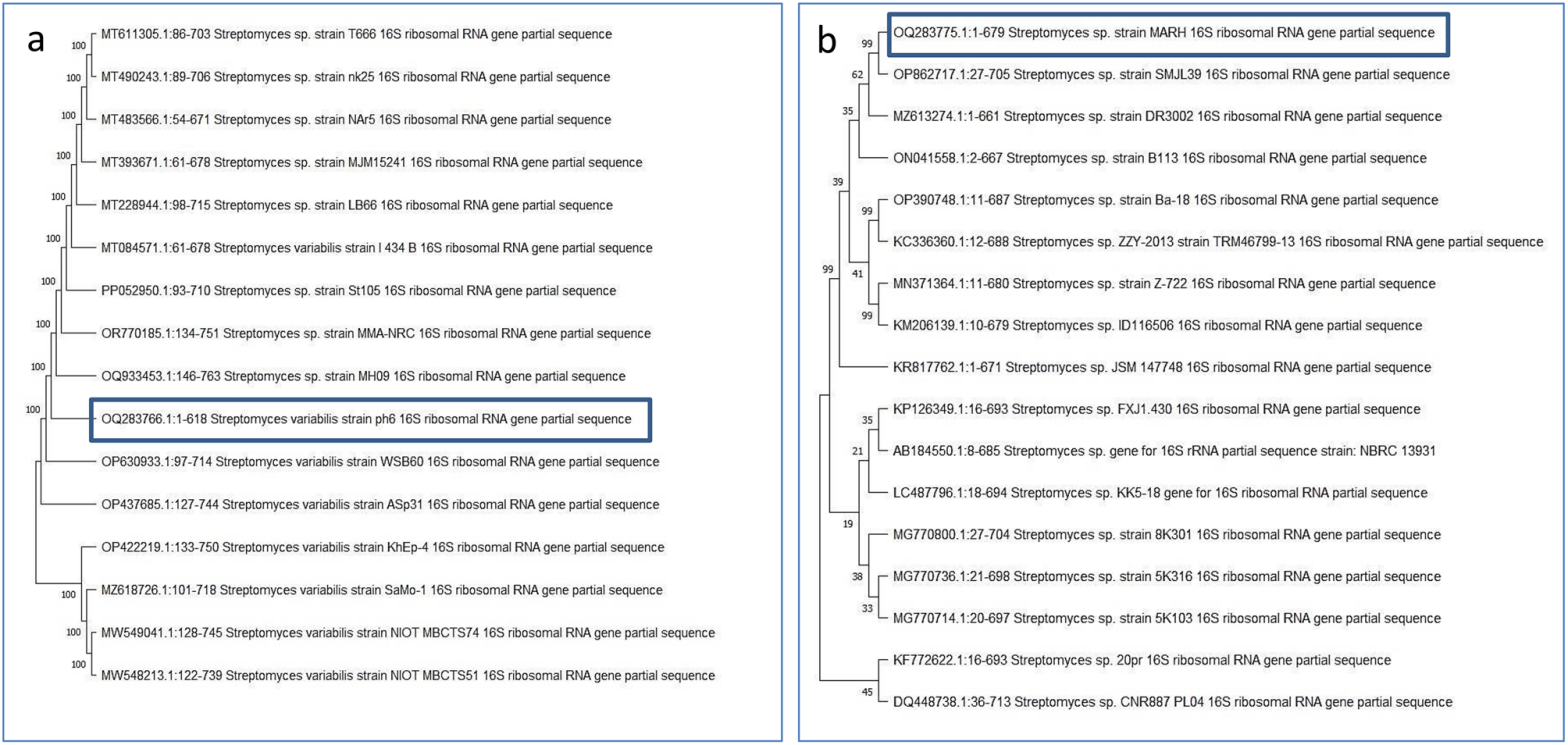
(**a**) Phylogenetic tree of *Streptomyces variabilis* and (**b**) *Streptomyces* sp. MARH.

The two most potent isolates (ph6 and MARH) with antiviral activities against CMV were subjected to morphological, physiological, and biochemical characteristics **(**Supplementary Table S1**, **Supplementary Table S2**, and **Supplementary Fig. S1**)**. The sequencing results and blast analysis confirmed the *Streptomyces* isolates, which were identified as *Streptomyces variabilis* strain ph6 and *Streptomyces* sp. strain MARH. Fig. 1 a and b displays the phylogenetic trees representing the partial sequence of 16 s rRNA from the local strain ph6 and strain MARH, in relation to closely similar sequences identified in GenBank databases. The selection process involved identifying *Streptomyces* strains with the highest similarity to our isolate’s 16S rRNA gene, ranging from 92 to 100%. These selected sequences were then connected to construct a phylogenetic tree that accurately represents the relationships between these strains.

### CMV disease symptoms development

**Fig. 2:**
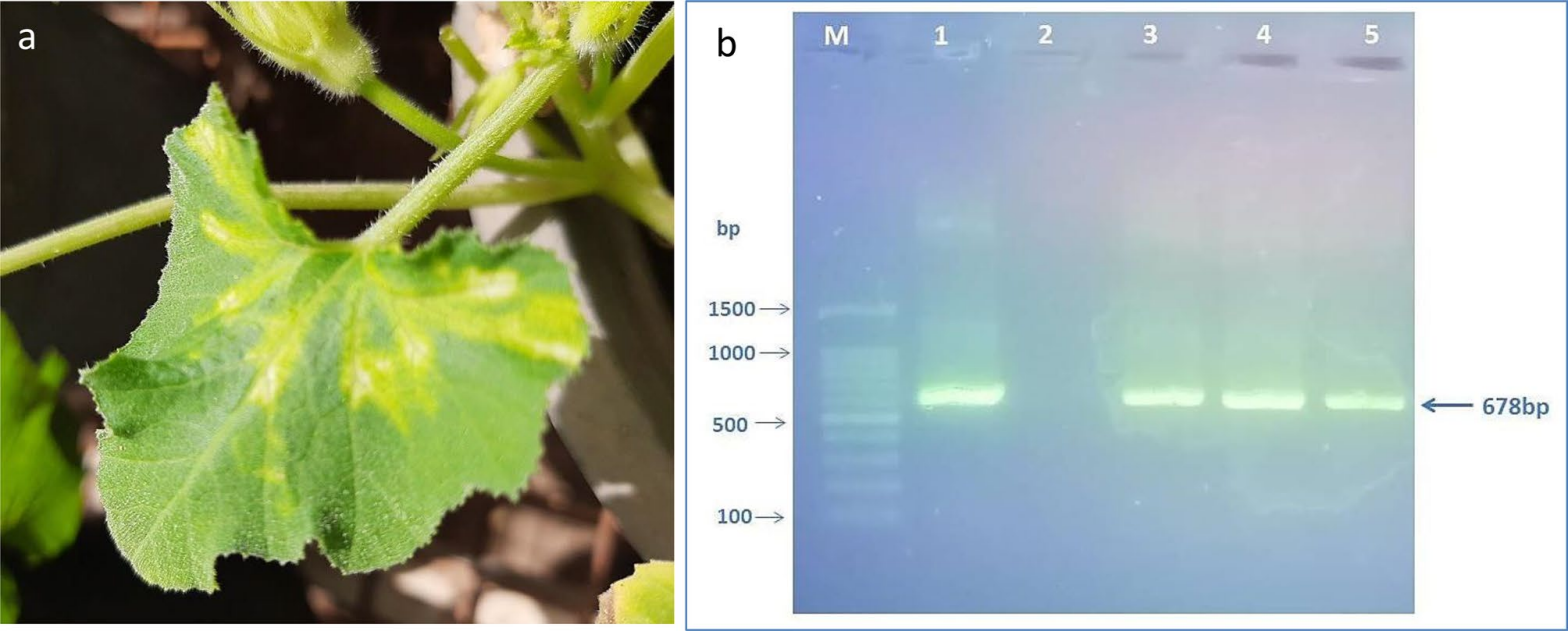
(**a**) Squash (*Cucurbita pepo* cv Eskandarani) leaves showing mosaic-like symptoms, chlorosis, vein banding, and leaf malformation following a systemic infection by CMV at 21 DPVI. (**b**) Amplified DNA fragments of the CMV obtained by RT-PCR with specific primers for the CMV-CP gene detected an amplicon of 678 bp (M, 100 bp ladder, 1 positive control, 2 negative control and 3, 4 and 5 CMV-infected squash plants). (DPVI) days post virus inoculation.

Twenty-one days after CMV inoculation, as well as after full growth of plants, plants inoculated with CMV showed typical viral infection symptoms compared to non-inoculated plants (healthy). CMV-infected squash plants exhibited systemic severe mosaic, chlorosis, and leaf deformation **(**Fig. 2a**)**.

### Detection of CMV using RT-PCR

RT-PCR with specific primers was used for detection of the CMV-CP gene with an amplified PCR product of 678 bp from infected squash plants, as shown in Fig. 2b and Supplementary Fig. S2**.**

### Sequence analysis and phylogenetic tree

The sequence of the CP gene was deposited to NCBI GenBank under accession number PX310541. Phylogenetic tree of our CMV isolate shared range of nucleotide sequence identity between 98%- 100% for CP gene with the other isolates published in the GenBank (Supplementary Fig. S3). Sequence analysis showed 100% nucleotide sequence identity with the Egyptian isolate OM891555 which classified into subgroup IA.

### Reactions of different plant species to CMV infection

**Fig. 3:**
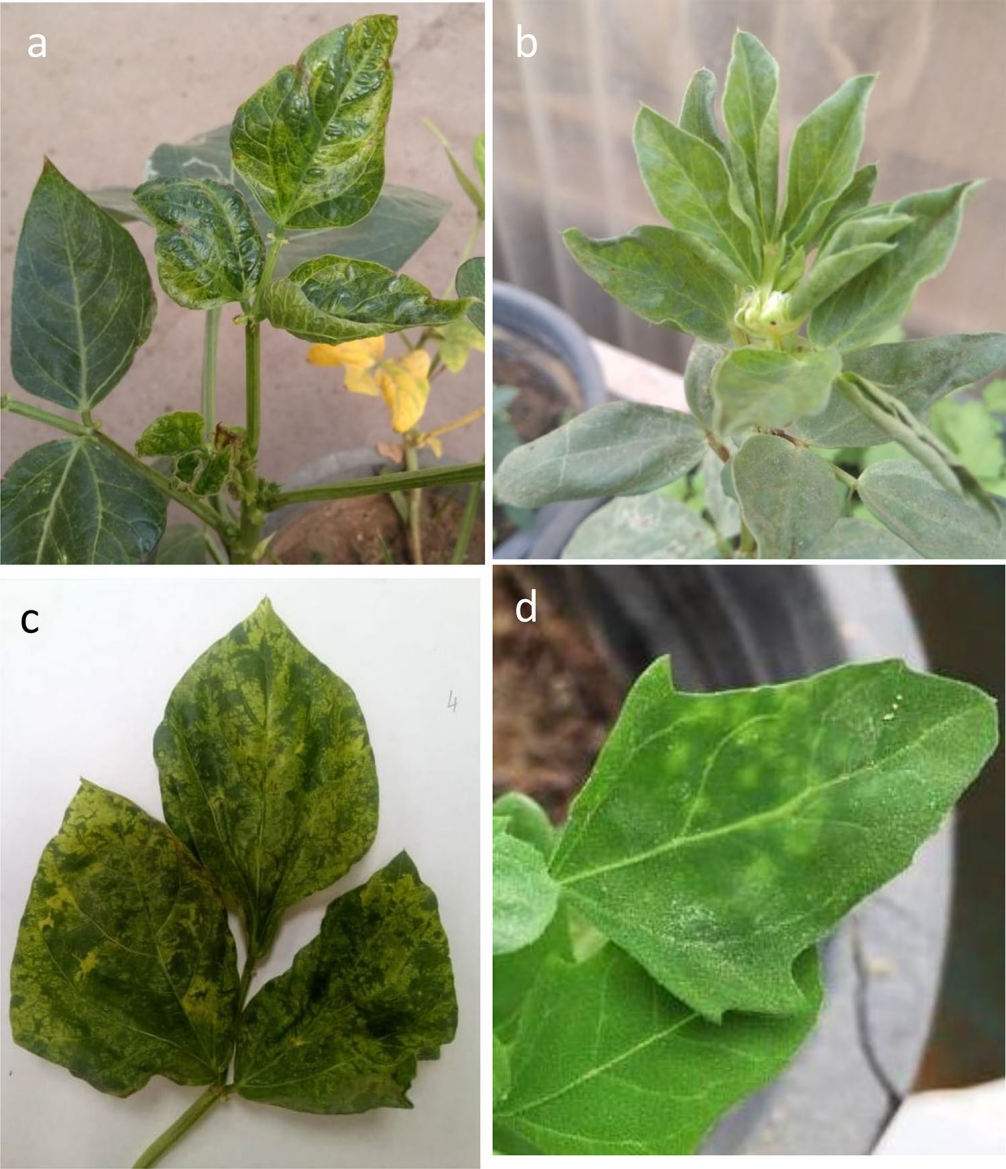
Reactions of different plant species to mechanical inoculation with CMV isolate (**a**) bean (*Phaseolus vulgaris*), (**b**) Faba bean (*Vicia faba* L.), (**c**) Cowpea (*Vigna unguiculata*) and (**d**) *Chenopodium amaranticolor.*

Response of four plants species belonging to two families to CMV infection is shown in **(**Fig. 3**).** The isolate CMV (PX310541) reacted positively with four plants. Distinct systemic symptoms were produced on the inoculated plants. Whereas, *Phaseolus vulgaris*, *Vicia faba* L and *Vigna unguiculata* plants gave severe mosaic, leaf deformation, mosaic and leaf curl and severe mosaic respectively. While the virus induced chlorotic local lesions on the inoculated leaves of *Chenopodium amararticolor*.

### Disease incidence and severity (%)

**Fig. 4:**
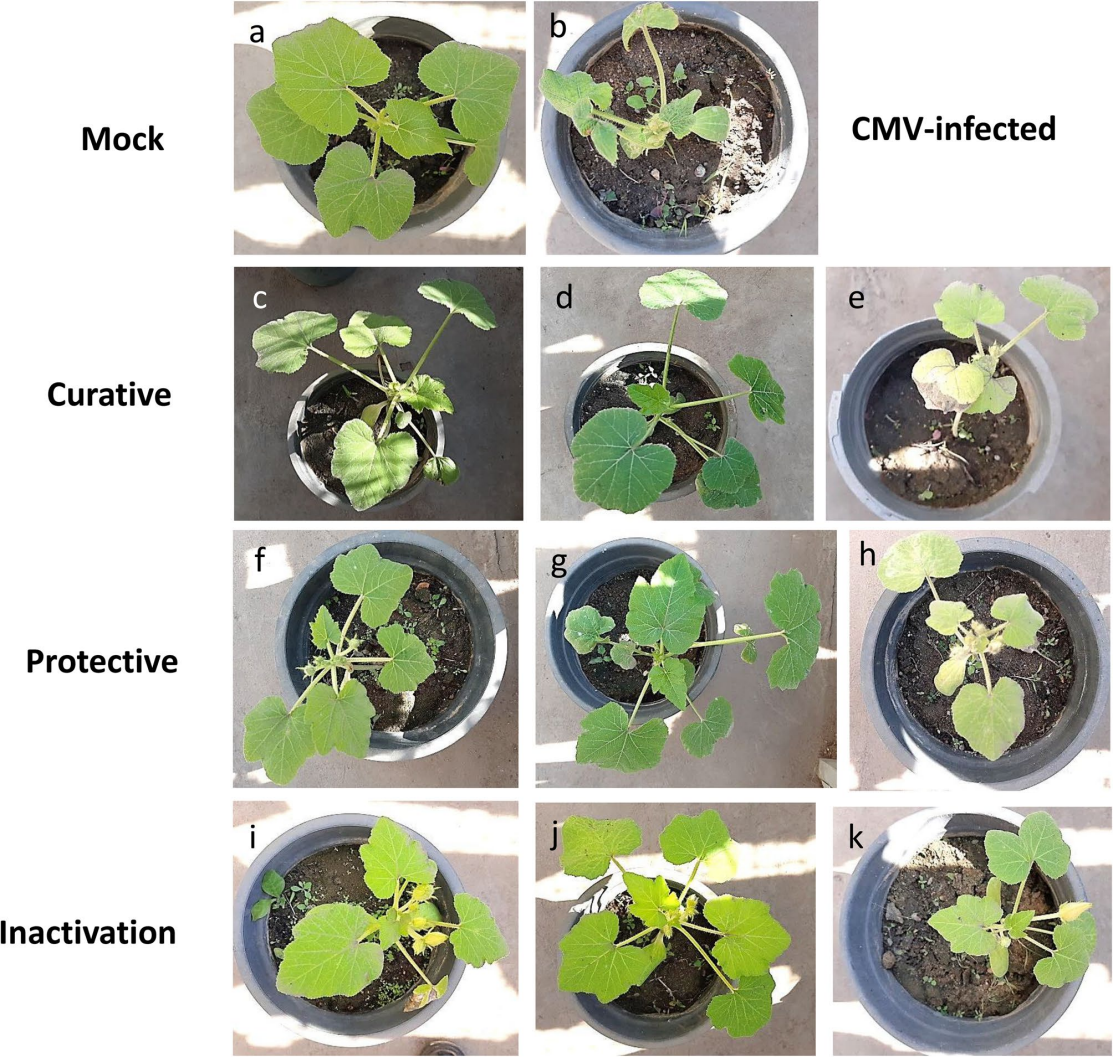
Comparison of CMV disease symptoms in response to SE1 and SE2 by different treatments (curative (C), protective (P) and inactivation (I)), (**a**) Mock with no symptoms on squash (*Cucurbita pepo* cv Eskandarani), (**b**) CMV infected showing severe mosaic and leaf malformation. (**c, d, e**) sprayed with C: SE1, C: SE2 and C: DMSO 24 h after CMV inoculation respectively, (**f, g, h**) sprayed with P: SE1, P: SE2 and P: DMSO 24 h before CMV inoculation respectively. (**i, j, k**) rubbed with the mixture of CMV + I: SE1, CMV + I: SE2 and CMV + I: DMSO respectively. (SE1) and (SE2) are bioactive crude extracts from *Streptomyces variabilis* and *Streptomyces* sp. MARH respectively.

The use of ethyl acetate extracts from *Streptomyces* spp. as curative (C), protective (P), or inactivation (I) treatments caused reduction or prevention of CMV infection in squash plants according to external disease symptoms and DAS-ELISA. Infected control plants had the greatest recorded rate of CMV infection (100%), as well as the highest disease severity (85 ± 2%). Mild mosaic and chlorosis symptoms appeared at 21 DPVI on certain plants undergoing curative and inactivation treatments, respectively, but no viral symptoms were evident on plants receiving protective treatment **(**Fig. 4**)**.

Treatments with P: SE1 and P: SE2 resulted in disease suppression of 100%, whereas P: DMSO solution resulted in suppression of only 31% compared to mock-inoculated plants. Curative treatments with C: SE1 and C: SE2 reduced CMV infection by 87% and 100%, respectively, while C: DMSO solution reduced it by about 35% only compared to mock-inoculated plants **(**Table 3**)**.

**Table 3:** Effect of curative, protective and inactivation treatments of *Streptomyces* spp. crude extract (SE1 and SE2) on virus concentration, disease incidence (%) and disease severity (%) for squash plants under CMV infection.

**Type of Treatment**	**Treatment group**	**Disease symptoms**	**Disease incidence (%)**	**Disease severity (%)**	Virus Concentration*
Negative control	Mock	No symptoms	0 ± 0^h^	0 ± 0^g^	0.181 ± 0.01^h^
Positive control	CMV-infected	Severe mosaic and leaf deformation	100 ± 0^a^	85 ± 2^a^	0.854 ± 0.00^a^
Curative	C: SE1	Mild mosaic or No symptoms	13 ± 1^g^	15 ± 1^e^	0.236 ± 0.02f.
	C: SE2	No symptoms	0 ± 0^h^	0 ± 0^g^	0.211 ± 0.01^fg^
	C: DMSO	Mosaic and yellowing	65 ± 3^c^	23 ± 2^d^	0.572 ± 0.01^b^
Protective	P: SE1	No symptoms	0 ± 0^h^	0 ± 0^g^	0.191 ± 0.02^gh^
	P: SE2	No symptoms	0 ± 0^h^	0 ± 0^g^	0.187 ± 0.0^gh^
	P: DMSO	Mosaic	69 ± 3^b^	52 ± 2^b^	0.537 ± 0.02^c^
Inactivation	I: SE1	chlorosis, and vein banding	30 ± 2^e^	13 ± 1^e^	0.271 ± 0.03^e^
	I: SE2	chlorosis	20 ± 2f.	10 ± 2f.	0.222 ± 0.01f.
	I: DMSO	Mild mosaic	40 ± 1^d^	33 ± 3^c^	0.341 ± 0.00^d^

* After 21 days post virus inoculation (DPVI) using DAS-ELISA, means (± SE standard error) followed by different letters are significantly different from each other as indicated by F-test among means Duncan’s multiple range test (p < 0.05). (C) Curative, (P) Protective and (I) inactivation treatments; (SE1) and (SE2) bioactive crude extracts from *Streptomyces variabilis* and *Streptomyces* sp. MARH respectively.

I: SE1 and I: SE2 inactivation treatments decreased CMV infection by approximately 70% and 80%, respectively, whereas I: DMSO solution decreased the infection percentage by 60%. No symptoms were observed on mock-inoculated squash plants. According to the symptoms, the protective treatments with P: SE1 and P: SE2 reduced the disease severity by 100%, but the use of P: DMSO solution only reduced it by 48%. Furthermore, utilizing C: SE1 and C: SE2 treatments decreased the severity of the viral disease by 85% and 100%, respectively, but by only about 77% when using the C: DMSO solution. In addition, I: SE1 and I: SE2 decreased the severity of the disease by 87% and 90%, respectively, while it decreased by around 67% when using the I: DMSO solution.

### Quantitative detection of CMV concentration by DAS-ELISA

Treatments of *Streptomyces* spp. crude extracts significantly decreased or suppressed CMV, as evidenced by a decrease in viral concentrations in newly produced squash leaves when compared to the untreated control (CMV-infected) using the DAS-ELISA method. When all squash plants were foliar-sprayed with *Streptomyces* spp. crude extracts (SE1 and SE2), through curative and protective methods, the CMV systemic concentration in leaves was significantly lower than in the infected control plants after 21 DAVI **(**Table 3**)**. Moreover, inactivation treatments (I: SE1 and I: SE2) reduced the optical density (O.D.) values of treated plants compared to the infected control.

In general, all asymptomatic squash plants treated with SE1 and (SE2 showed no CMV incidence in squash leaves detected by the DAS-ELISA assay, indicating the accumulation of CMV in the treated leaves might be inhibited, particularly in the systemic leaves of the plants. The treatments with DMSO solution had a minimal effect on CMV content in the treated plants compared to the effects of SE1 and SE2 treatments. The most significant impact was seen when using the protective treatment with SE1 and SE2, along with curative and inactivation **(**Table 3**)**.

### Effect of crude extracts of Streptomyces spp. on photosynthetic pigments

**Fig. 5:**
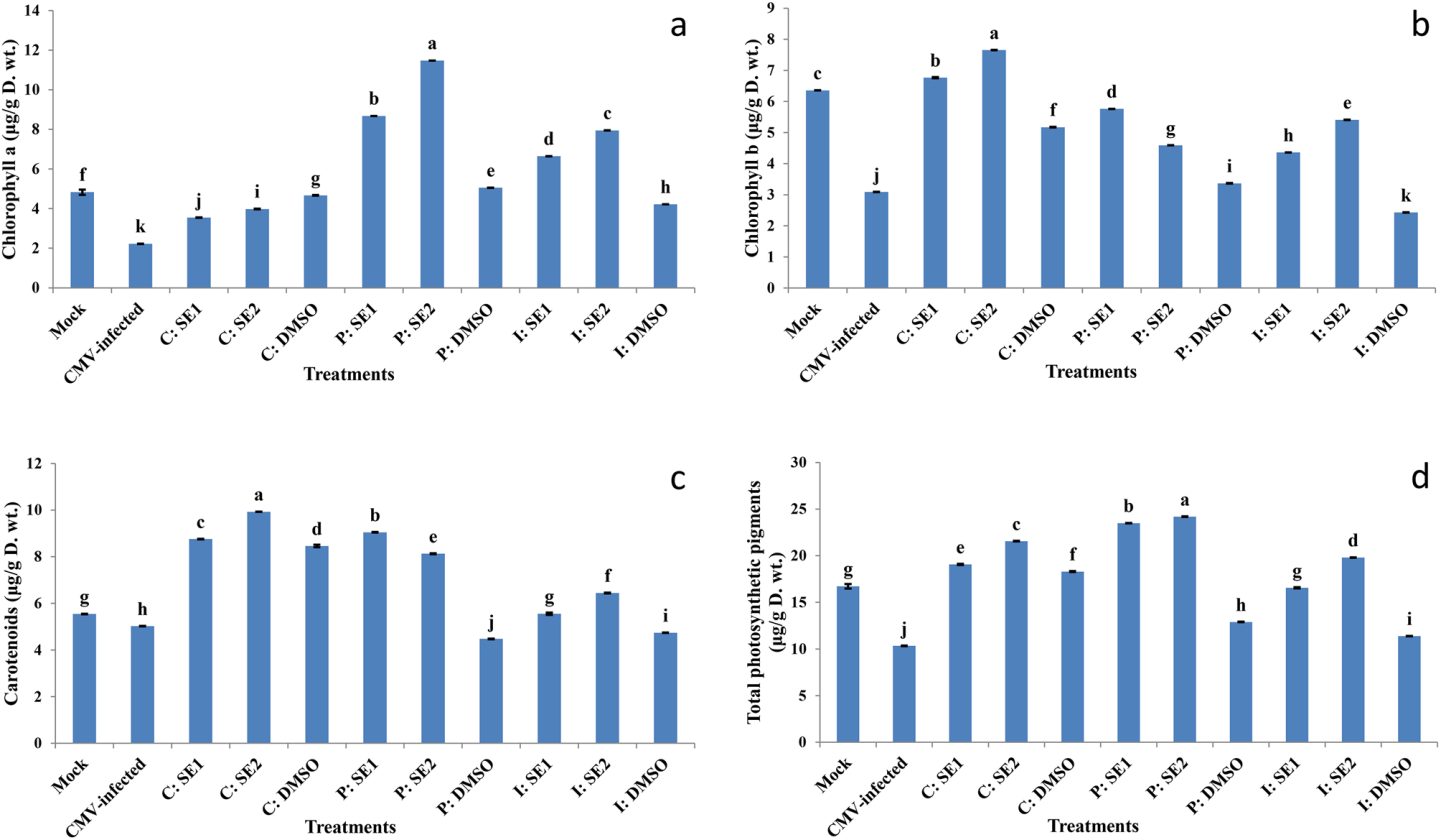
Effect of application of SE1 and SE2 through curative, protective and inactivation methods on photosynthetic pigments (**a–d**) content in leaves of squash plant under healthy control (mock) and CMV infection (CMV-infected) after 21 days of virus inoculation. The data were expressed as mean ± standard error (SEM). Means with the different letters (a, b, c, d, e, f, g, h, i, j, k) are significantly different at the 0.05 level among treatments according to F-test. (SE1) and (SE2) are bioactive crude extracts from *Streptomyces variabilis* and *Streptomyces* sp. MARH respectively.

For CMV-infected squash plants, the virus caused a significant reduction in chlorophyll a (54.01%), chlorophyll b (51.39%), carotenoid (9.34%), and total photosynthetic pigment contents (38.2%), compared to mock-inoculated plants **(**Fig. 5**).** Additionally, by using curative, protective, and inactivation techniques, SE1 and SE2 mitigated the severe effects of CMV on chlorophyll contents and improved chlorophyll concentration. The P: SE2 method was the most effective treatment for increasing photosynthetic pigment contents, which increased the total chlorophyll by 57.26% in the leaves of squash plants over CMV-infected plants **(**Fig. 5**)**.

### Effect of crude extracts of Streptomyces spp. on antioxidant enzymes (APX and CAT)

**Fig. 6:**
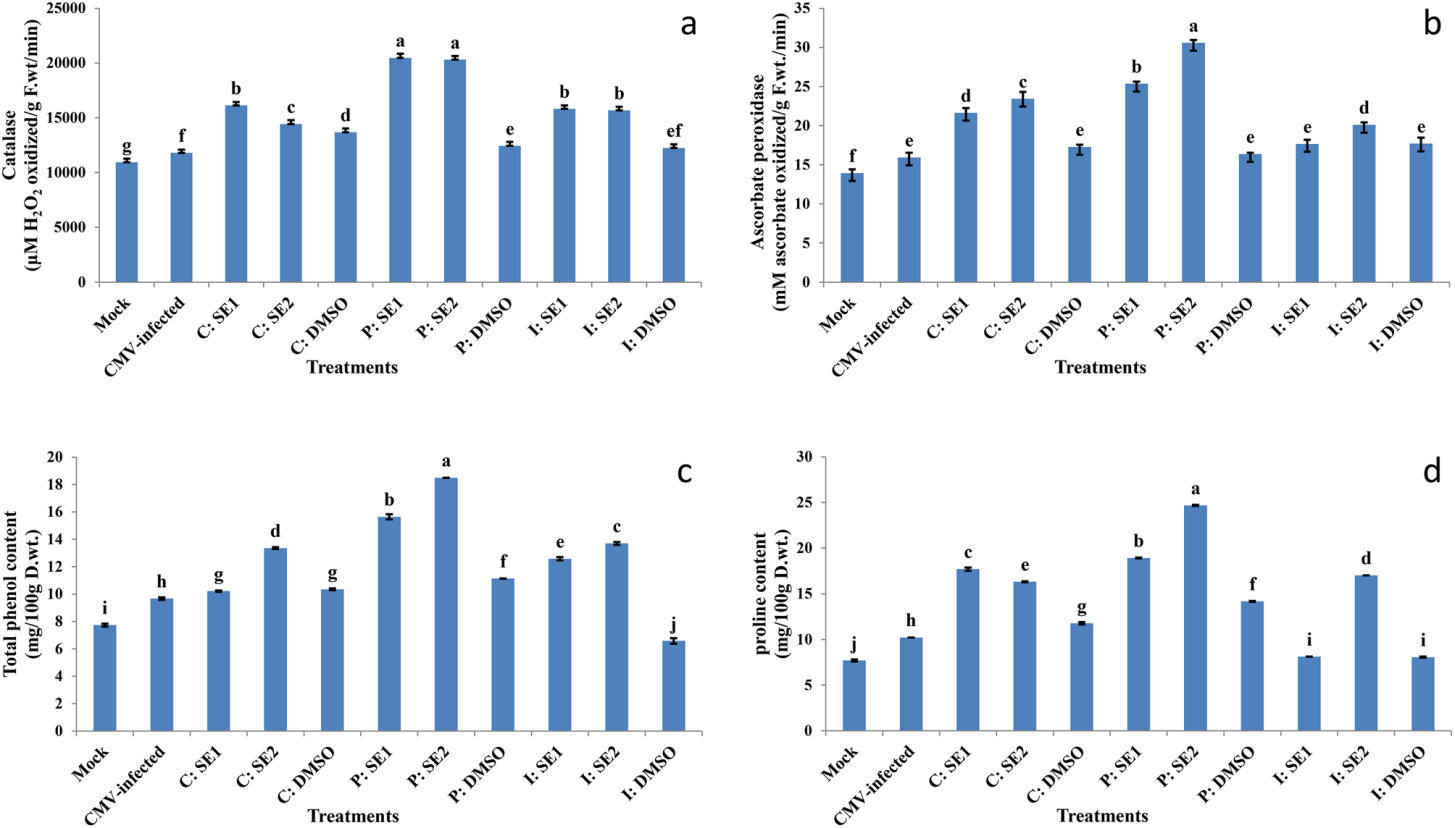
Effect of application of SE1 and SE2 through curative, protective and inactivation methods on (**a**) Catalase (CAT), (**b**) Ascorbate peroxidase (APX) activity, (**c**) total phenol and (**d**) proline content in leaves of squash plant under healthy control (mock) and CMV infection (CMV-infected) after 21 days of virus inoculation. The data were expressed as mean ± standard error (SEM). Means with the different letters (a, b, c, d, e, f, g, h, i, j) are significantly different at the 0.05 level among treatments according to F-test. (SE1) and (SE2) are bioactive crude extracts from *Streptomyces variabilis* and *Streptomyces* sp. MARH respectively.

At 21 DPVI, CMV infection slightly increased catalase (CAT) activity compared to the mock-inoculated plants (Fig. 6a). The curative treatments (C: SE1 and C: SE2) enhanced the activity of CAT by 26.92% and 18.24%, respectively, when compared to the CMV-infected control plants. The CAT activity was higher in the P: SE1 and P: SE2 (42.38% and 41.91%) compared to the infected control plants, respectively. The inactivation treatment (I: SE1 and I: SE2) resulted in a 25.45% and 24.80% increase in CAT activity, respectively, when compared to the infected control plants. Treatments with DMSO solution slightly increased CAT activity compared to mock-inoculated plants, but decreased compared to the three treatments **(**Fig. 6a**)**.

Ascorbate peroxidase (APX) activity was elevated by CMV infection at 21 DPVI in comparison to the mock-inoculated plants (Fig. 6b). Furthermore, the C: SE1 and C: SE2 enhanced the activity of APX by 26.35% and 32.11%, respectively, when compared to the infected control plants. The P: SE1 and P: SE2 increased APX activity (37.20% and 47.89%) in comparison to infected control plants. Inactivation treatments (I: SE1 and I: SE2) increased the activity of APX (9.84% and 20.74%) compared to the CMV-infected plants, respectively. In comparison to mock-inoculated plants, treatments with DMSO solution faintly increased the enzyme activity.

### Effect of crude extracts of Streptomyces spp. on total phenol and proline content

At 21 DPVI, CMV infection increased the phenol content as compared to the mock squash plants **(**Fig. 6c**)**. In addition, treated plants with extracts (SE1 and SE2) had higher phenol content than infected control plants in the three treatments; the maximum value of phenol content was detected in protectively treated plants with P: SE1 and P: SE2 by 38.18% and 47.72% respectively compared to the CMV-infected plants.

At 21 DPVI, CMV infection increased the proline content when compared to the mock-inoculated plants **(**Fig. 6d**)**. But treatments of plants with SE1 and SE2 increased the proline content through protective, curative, and inactivation methods as compared to the CMV-infected plants, but the higher contents of proline were detected in P: SE1 and P: SE2 by 45.99% and 58.61% respectively relative to the CMV-infected plants.

### Effect of crude extract of Streptomyces spp. on membrane stability and electrolyte leakage

**Fig. 7:**
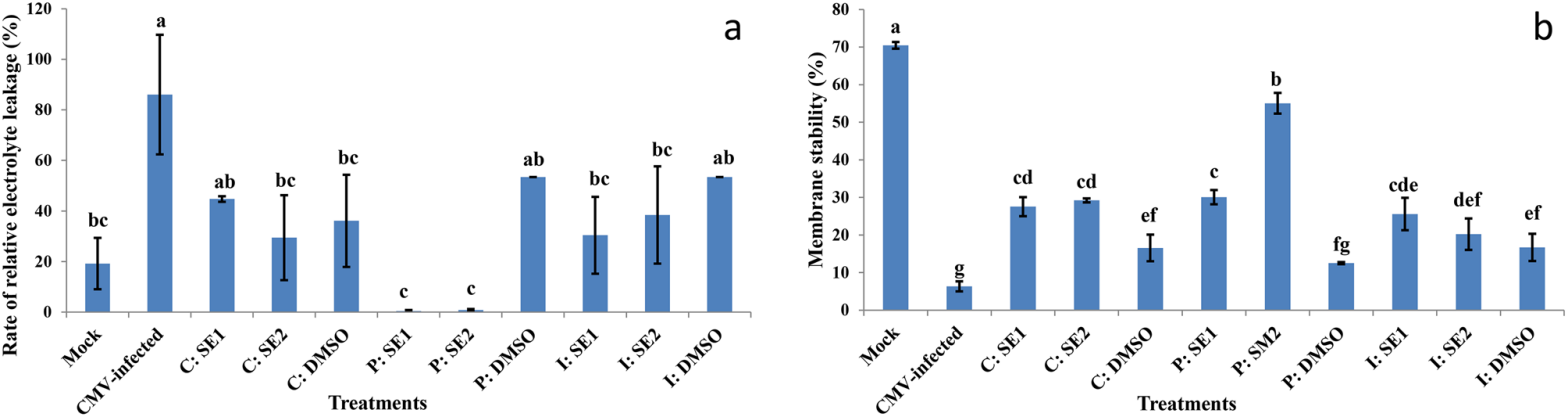
Effect of application of SE1 and SE2 through curative, protective and inactivation methods on (**a**) relative electrolyte leakage rate (EL) (%) and (**b**) membrane stability index (MSI) (%) in leaves of squash plant under healthy control (mock) and CMV infection (CMV-infected) after 21 days of virus inoculation. The data were expressed as mean ± standard error (SEM). Means with the different letters (a, b, c, d, e, f, g) are significantly different at the 0.05 level among treatments according to F-test. (SE1) and (SE2) are bioactive crude extracts from *Streptomyces variabilis* and *Streptomyces* sp. MARH respectively.

Relative electrolyte leakage rate in the CMV-infected plants increased by 77.66% compared with non-infected plants (Mock) at 21 DPVI, and also was significantly higher than that in the curative method (47.99% and 65.77%), in the protective method (99.53% and 99.05%), and in the inactivation method (64.65% and 55.32%) by SE1 and SE2, respectively **(**Fig. 7a**)**. In addition, the relative electrolyte leakage rate in the DMSO-treated plants increased by 46.76%, 64.02%, and 64.02% compared with those non-infected plants (Mock) in curative, protective, and inactivation methods respectively.

Membrane stability in the CMV-infected plants decreased by 91% compared with the non-infected plants (Mock). Membrane stability in SE1- and SE2 treated plants was significantly higher than in CMV-infected plants through the curative method (77.00% and 78.32%), the protective method (78.93% and 88.49%), and the inactivation method (75.22% and 68.66%), respectively **(**Fig. 7b**)**. In addition, membrane stability in the DMSO-treated plants in curative, protective, and inactivation methods decreased by 76.53%, 82.23%, and 76.32% compared with those non-infected plants (Mock), respectively.

### Effect of crude extracts of Streptomyces spp. on pathogenesis-related genes (PR-b1) and (PR-2) expression under CMV challenge

**Fig. 8:**
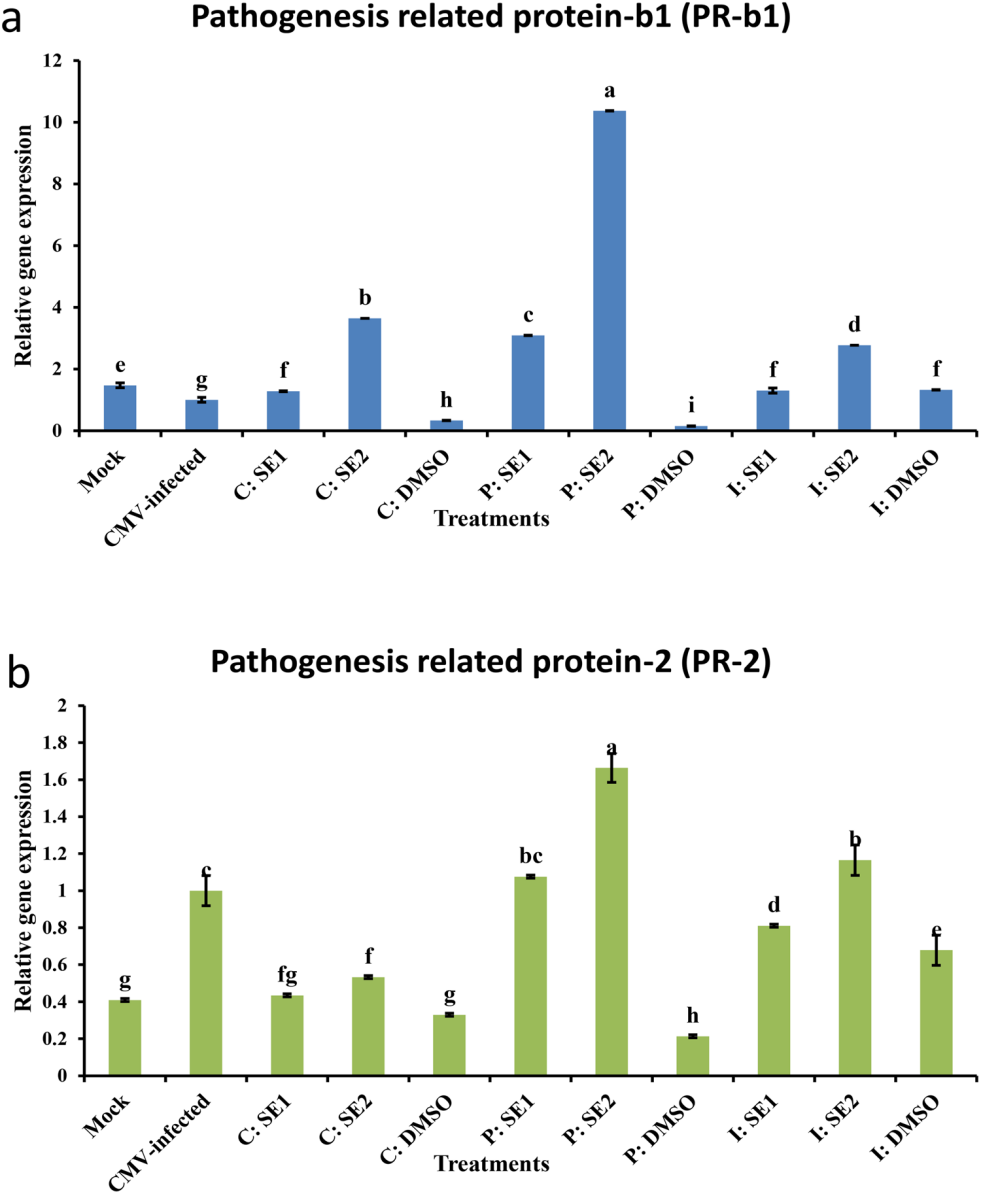
Relative expression levels of (**a**) pathogenesis-related protein gene (PR-b1) and (**b**) pathogenesis-related protein gene (PR-2) in squash plants after application of SE1 and SE2 through curative, protective and inactivation methods under healthy control (mock) and CMV infection (CMV-infected) after 21 days of virus inoculation. The data were expressed as mean ± standard error (SEM). Means with the different letters (a, b, c, d, e, f, g, h, i) are significantly different at the 0.05 level among treatments according to F-test. (SE1) and (SE2) are bioactive crude extracts from *Streptomyces variabilis* and *Streptomyces* sp. MARH respectively.

Plants typically exhibit high levels of multiple pathogenesis-related (PR) genes that control their response to various abiotic and biotic stresses. PR-1 and PR-2 are essential in viral infection, alongside other factors. After 21 DPVI for all treatment groups (curative, protective, and inactivation) treated with all strains of *Streptomyces* spp*.*, squash plants showed different gene expression of PR-b1 and PR-2 genes compared to untreated squash plants (CMV-infected control) **(**Supplementary Fig. S4**)**. As shown in Fig. 8a, in the crude extract-treated groups, the PR-b1 gene expression was upregulated to 1.28- and 3.64-fold increases in C: SE1 and C: SE2 respectively compared to untreated squash plants (CMV-infected control).

PR-b1 overexpression, reporting about 3.09- and 10.37-fold increases in protective methods compared to the CMV-infected squash plants for P: SE1 and P: SE2, respectively. In inactivation methods, PR-b1 gene expression was increased about 1.3-fold by I: SE1. While in the I: SE2-treated groups, the PR-b1 gene expression reported about a 2.77-fold increase compared to the CMV-infected treatment group.

Regarding the PR-2, the results indicated a down-regulation of PR-2 gene expression of about 0.43 and 0.53 in C: SE1 and C: SE2 respectively compared to CMV-infected control.

PR-2 overexpression was reported to have about 1.66-fold increases in protective methods compared to the CMV-infected squash plants for P: SE2. Additionally, PR-2 gene expression was upregulated by approximately 1.07-fold compared to the CMV-infected control group treated with P: SE1.

In the inactivation method, PR-2 gene expression was downregulated by about 0.81 by I: SE1, while PR-2 overexpression was reported to have about 1.16-fold increases by I: SE2 relative to the CMV-infected treatment group **(**Fig. 8b**)**.

### Identification of bioactive compounds in the ethyl acetate crude extracts of Streptomyces spp. using GC–MS

The ethyl acetate crude extracts were found to be effective in inhibiting or controlling CMV through curative, protective, and inactivation methods. These effects might be related to a bioactive component in extracts of *Streptomyces* spp.

GC–MS fractionation revealed eight and ten chemical compounds with different molecular weights for strain ph6 and strain MARH, respectively. Among these compounds, four had antiviral activities. Table 4 and Table 5 list the total peak area of the compounds that were detected, as well as the retention time, molecular weight, molecular formula, structures, and biological activity of the identified compounds.

**Table 4:** GC mass profile of ethyl acetate extract from *Streptomyces variabilis* (SE1).

No	RT	Probability	Compound Name	Area %	MW	MF	The chemical structure	Biological activity
1	5.24	21.30	1­Pentanol, 4­amino­	0.27	103	C_5_H_13_NO		
2	5.67	21.53	Thr­Val­Lys	2.56	346	C_15_H_30_N_4_O_5_		Antioxidant ^96^
3	5.78	16.49	1,3­Pentanediol, 2­methyl­, 1­propanoate	1.98	174	C_9_H_18_O_3_		Anti-inflammatory and anti-tumor ^97^
4	5.88	15.36	3­Pentanethiol, 2­methyl­ (CAS)	2.17	118	C_6_H_14_S		
5	6.37	42.60	1,3­Dinitro­2­imidazolidinone	0.69	176	C_3_H_4_N_4_O_5_		Antiviral ^90^
6	6.50	23.69	(R)­(­)­2­Ethylhexan­1­ol	0.57	130	C_8_H_18_O		
7	8.84	66.57	2­Chloropentylacetate	1.26	164	C_7_H_13_ClO_2_		
8	9.10	32.57	L­Aspartic acid	0.32	133	C_4_H_7_NO_4_

**Table 5:** GC mass profile of ethyl acetate extract from *Streptomyces* sp. MARH (SE2).

No	RT	Probability	Compound Name	Area %	MW	MF	The chemical structure	Biological activity
1	11.59	11.43	Dichloroacetaldehyde	0.76	112	C_2_H_2_Cl_2_O		Antibacterial ^98^
2	12.62	6.22	Trans-2-phenyl-1,3-dioxolane-4-methyl octadec-9,12,15-trienoate	0.78	440	C_28_H_40_O_4_		Antioxidant and Anti-cancer ^99^
3	13.01	76.18	Nephthoside—1,2’,3’,4’-Tetraacetate	0.82	696	C_40_H_56_O_10_		Antiviral ^62^
4	16.78	11.94	Pyrrolizin-1,7-dione-6-carboxylic acid, methyl(ester)	0.78	197	C_9_H_11_NO_4_		Antitumor and Antiviral ^100^
5	17.74	65.97	1-(o-Carboran-9-yl)−2,3,4,5,6-pentaphenylbenzene	0.75	602	C_38_H_36_B_10_		
6	17.82	17.01	L-Lysine	1.50	146	C_6_H_14_N_2_O_2_		Antiviral ^88^
7	19.05	28.62	Dodecane, 2,2,4,9,11,11-hexamet	2.30	254	C_18_H_38_		Antibacterial ^101^
8	19.48	12.42	Tetraphenylporphyrinato dichlorotitanium(IV)	0.73	730	C_44_H_28_C_l2_N_4_Ti		Anti-cancer ^102^ and Antiviral ^103^
9	20.40	21.48	Bis(3,6,9,12-tetraoxapentaethylene)crowno-N,N,N’,N’-tetramethyl-p-phanediamine	0.75	765	C_40_H_68_N_4_O_10_		
10	20.91	11.20	2-Myristynoyl pantetheine	0.78	484	C_25_H_44_N_2_O_5_S		

**Fig. 9:**
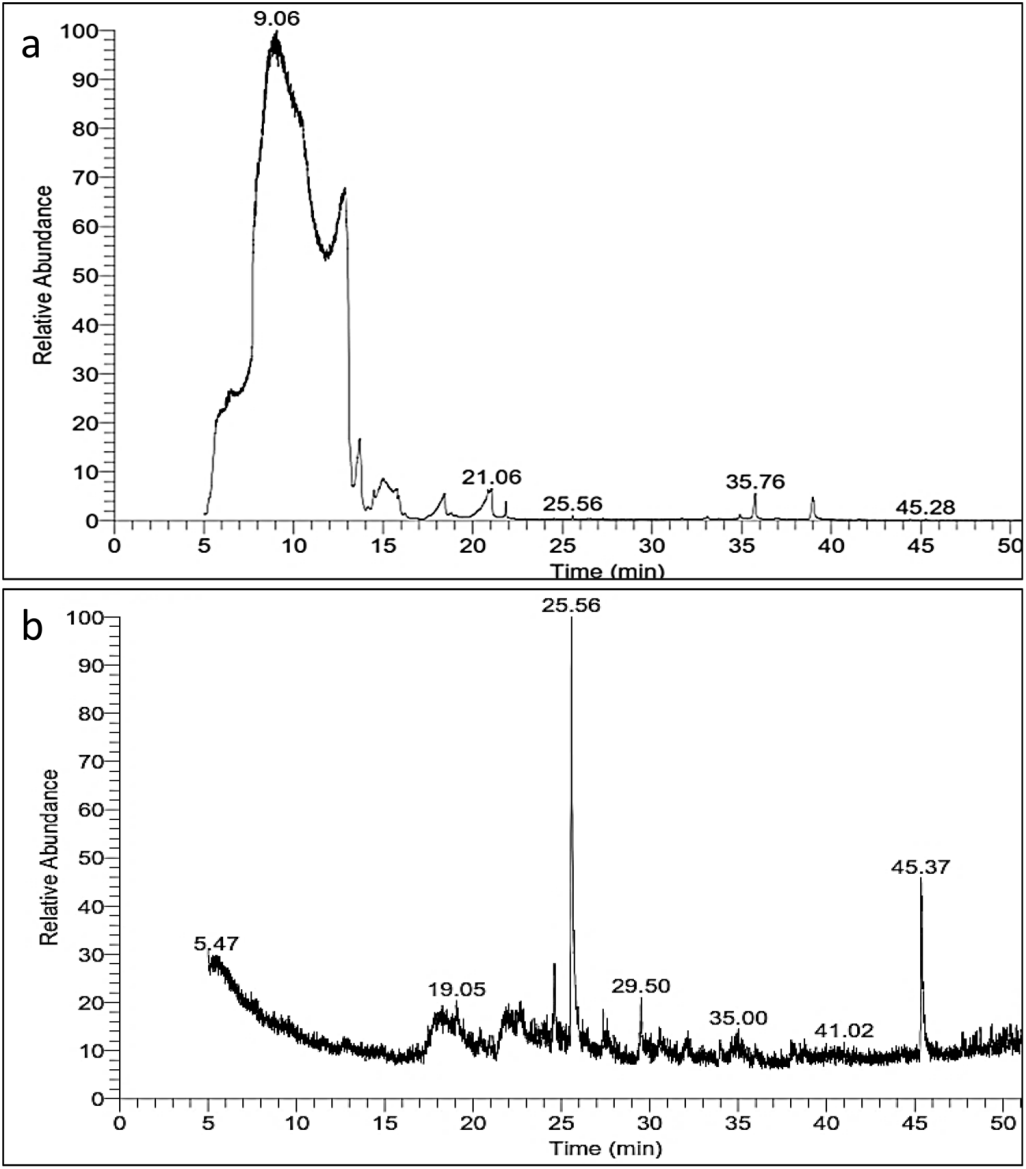
GC–MS chromatogram of the ethyl acetate extracts from (**a**) *Streptomyces variabilis* and (**b**) *Streptomyces* sp. MARH.

The chromatogram of extract from strain ph6 is shown in Fig. 9a, and the compounds were 1Pentanol,4amino, ThrValLys, 1,3Pentanediol,2methyl,1propanoate, 3Pentanethiol,2methyl (CAS), 1,3Dinitro2imidazolidinone, (R)()2Ethylhexan1ol, 2Chloropentylacetate, LAspartic acid **(**Table 4**)**.

The chromatogram of extract from strain MARH is shown in Fig. 9b, and the compounds were Dichloroacetaldehyde, Trans-2-phenyl-1,3-dioxolane-4-methyl octadec-9,12,15-trienoate, Nephthoside-1,2’,3’,4’-Tetraacetate, Pyrrolizin-1,7-dione-6-carboxylic acid, methyl(ester), 1-(o-Carboran-9-yl) −2,3,4,5,6-pentaphenylbenzene, L-Lysine, Dodecane, 2,2,4,9,11,11-hexamet, Tetraphenylporphyrinato dichlorotitanium(IV), Bis (3,6,9,12-tetraoxapentaethylene) crowno-N,N,N’,N’-tetramethyl-p-phanediamine, 2-Myristynoyl pantetheine **(**Table 5**)**.

## Discussion

Plant viruses are a major threat to food security due to their devastating impact on crops. *Cucumber mosaic virus* (CMV) is one of the most widespread plant viruses and cause significant economic losses in many vegetable and horticultural crops. In the current study, biological and molecular detection using RT-PCR was used to identify CMV isolate with an accession number PX310541. According to numerous researchers, CMV frequently causes a wide range of symptoms, such as mosaic, reduced leaf size, stunted growth, and yield losses^45^. The widespread use of agrochemicals to suppress the virus’s spread and control the transmission vector leads to contamination of the environment, and pesticide-resistant aphids. Thus, the previously noted issues with managing viral infections have sparked a renewed interest in biological control as a substitute approach to disease management. *Streptomyces* species are now a safe, effective, and sustainable way to treat infections. *Streptomyces* secondary metabolites play a critical role in crop protection as alternative bioproducts to chemical pesticides and fertilizers^46,47^. In this study, two novel *Streptomyces* spp. were isolated from marine aqua systems and molecularly identified as *Streptomyces variabilis* strain ph6 and *Streptomyces* sp. strain MARH with accession numbers OQ283766 and OQ283775 respectively. The two promising strains produced novel bioactive compounds **(**Tables 4 and 5**)** as secondary metabolite products. *Streptomyces* spp. possess an average of about 25–70 biosynthetic gene clusters (BGCs) encoding secondary metabolite products with a high diversity of bioactive compounds from one strain^48–50^. The antiviral potential of crude extracts (SE1 and SE2) derived from *Streptomyces variabilis* and *Streptomyces* sp. MARH respectively was evaluated using the disease severity (DS), disease incidence (DI), photosynthetic pigments, viral accumulation, antioxidant enzyme activity, and pathogenesis-related proteins (PRs) in the treated infected squash plants through curative, protective and inactivation methods. The application of *Streptomyces* extracts (SE1 and SE2) led to the effective control of CMV in squash plants under greenhouse conditions. *Actinomycetes* which were isolated from a marine habitat had antiviral activity against CMV^51^. *Streptomyces* can inhibit viral infections in crops by generating secondary metabolites and enzymes^52^. According to Mohammed et al.^53^, potato cultivars treated with the filtrate from *Streptomyces fradiae* QD3 showed decreased infection rates, disease severity, and PVY optical density. *Streptomyces* culture filtrate shows promise as a biocontrol agent against PVY in potatoes through enhanced plant defense activation and direct antiviral action^54^.

In our study, there was a 100% protection rate observed on squash plants pretreated with crude extract of strain MARH (SE2) and of strain ph6 (SE1). As well, Glycoprotein (GP-1) from *Streptomyces* sp. ZX01 had a higher protective efficacy (87.58%) than curative efficacy (13.44%) on *Nicotiana tabacum* against *Tobacco Mosaic Virus* (TMV) suggesting that the plants may have an immune-stimulating impact^9^.

Protective treatments (P: SE2 and P: SE1) showed the maximum inhibition of CMV symptoms and disease severity at 21 days post virus inoculation (DPVI) **(**Table 3**)**. In the same context, foliar spraying *Cucumis sativus* with *S. albovinaceus* and *S. sparsogenes*, separately, reduced the mosaic symptoms caused by the *Zucchini Yellow Mosaic Virus* (ZYMV) by 95% and 100%^55^.

Furthermore, the accumulation of CMV in squash leaves at 21 DPVI was detected using the DAS-ELISA assay^56,57^. Also, Taha et al.^58^ used indirect (I-ELISA) for demonstrating *Tomato mosaic virus* (ToMV) titer in tomato plants treated with culture filtrate (CF) of *S. ovatisporus* LC597360 and *S. rectiviolaceus*. Our results indicated that the P: SE2 and P: SE1 significantly decreased CMV accumulation titer in the leaves of squash with suppression of disease severity under greenhouse conditions. These results are in line with Abo-Zaid et al.^59^, who reported that foliar application of pellet suspension of *Streptomyces cellulosae* isolate Actino 48 decreased TMV accumulation in tomato plants with the reduction of symptoms and severity of the virus (21 dpi). Nasr-Eldin et al.^60^ indicated that potato plants treated with crude filtrates of *Streptomyces* spp. almost completely prevented *Potato virus Y* (PVY^NTN^) infection and reduced PVY^NTN^ concentration, and this was noticed by detecting the mean absorbance values of PVY^NTN^ in plant leaves using DAS-ELISA.

The inhibition of CMV infectivity can be attributed to the potential antiviral effect of bioactive compounds in *Streptomyces* spp. extracts (SE1) and (SE2), such as 1,3¬Dinitro¬2-imidazolidinone, Nephthoside-1,2’,3’,4’-Tetraacetate, Tetraphenylporphyrinato dichlorotitanium(IV), and L-Lysine, as they prevented the virus replication cycle^61^. The decrease in virus accumulation in squash plants can be attributed to the fact that the diterpenes/diterpenoids and their derivatives had high binding affinities toward the viral protein^62^. It appears that crude extracts (SE2 and SE1) have an antiviral effect by either directly inhibiting virus replication or indirectly by making the host plant more resistant to the virus on a systemic level^58^. Systemic acquired resistance (SAR) is marked by the build-up of salicylic acid (SA), increased expression of pathogenesis-related proteins (PRs), and activation of the phenylpropanoid pathway which results in the production of higher phenolic compounds.

In addition, squash plants infected with CMV had significantly lower amounts of chlorophyll a, b, and carotenoid compared to healthy control plants. There were similar results showing that CMV infection resulted in decreased levels of photosynthetic pigments and inhibited plant growth in tomatoes and cucumbers^63-65^. Decreased chlorophyll levels in virus-infected plants may result from the activation of particular cellular enzymes like chlorophyllase or from the virus’s impact on pigment production^66,67^. Treating squash plants with crude extracts (SE1 and SE2) using curative, protective, and inactivation methods resulted in increased chlorophyll content, suggesting that the bioactive substances in *Streptomyces* spp. extracts can eliminate the virus, ultimately boosting the plant’s ability to fight off disease.

Ascorbate peroxidase (APX) is one of the most significant antioxidant enzymes in the reactive oxygen metabolic pathway of plants. Furthermore, Catalase (CAT) also have a crucial role in safeguarding plant cells from reactive oxygen species (ROS)-induced oxidative damage after stress exposure^68^. CMV infection (infected control) increased the activities of antioxidant enzymes (APX and CAT) compared to those of healthy plants (mock). In contrast, squash plants treated with *Streptomyces* spp. extracts (SE1 and SE2) had increased APX and CAT activities compared to the infected control. This effect could be the result of increased expression of a specific defense gene. Culture filtrate (CF) of *Streptomyces ovatisporus* LC597360 induced resistance of tomato plant against *Tomato mosaic virus* (ToMV) by increasing the activity of CAT and APX in comparison with infected control^58^. On the other hand, Ningnanmycin is an antiviral substance extracted from *Strepcomces noursei* var. xichangensis may be responsible for improved resistance against TMV-infected plants by the induction of resistance-related enzymes such as peroxidase (POD), phenylalanine ammonia-lyase (PAL), and superoxide dismutase (SOD) activity^8,69^.

Plant defense against viruses has been closely linked to phenolic compounds. Treatment of squash plants with SE1 and SE2 through curative, protective, and inactivation methods increased the total of phenolic compounds compared to the infected control; the higher concentration of phenolic compounds was in protective-treated plants with SE1 and SE2 by 38.18% and 47.72%, respectively, compared to the infected control plants^70^.

The synthesis of lignin and suberin, which are known to be involved in the formation of physical barriers against the spread of diseases, may be facilitated by the higher concentration of phenolic compounds in the host plants, therefore strengthening the host cell walls^71^.

Proline has been linked to plants’ defense mechanisms against pathogens^72^. Proline accumulation level indicated significant lipid peroxidation and stress adaptation mechanism. Proline levels were elevated in *Arabidopsis thaliana* during plant resistance to pathogen attack^73,74^. In our experiment, proline levels were higher in CMV-infected squash plants than in the healthy controls^70,75,76^. In contrast, SE1- and SE2- treated plants had increased proline content relative to leaves from CMV-infected plants. But a higher amount of proline was detected in protective treatments (P: SE1 and P: SE2) by about 45.99% and 58.61%, respectively compared to the infected control plants. Reactive oxygen species (ROS) are released by plants exposed to microbial infections, and these ROS induce the plant cells surrounding the infection site to undergo programmed cell death. This procedure successfully walls off the pathogen and ends the disease process^77^. Proline has the potential to be a strong ROS scavenger, which could stop ROS from causing programmed cell death^78^.

Cell membrane damage is usually indicated by increased permeability in diseased plant tissues. Cell death was always preceded by electrolyte leakage, a sign of altered permeability. Oxygen-free radical-induced membrane lipid peroxidation may be the cause of the strong electrolyte leakage found in many diseased tissues^79^.

In our study, the relative electrolyte leakage rate in the CMV-infected plants (infected control) increased by 77.66% compared with non-infected plants (Mock). Increase in permeability in virus-infected leaves might be due to a general wound stimulus^80^. Applications of P: SE1 and P: SE2 protected plant cell membranes against CMV infection, and likewise, *Actinomycete* strains were found to provide superior protection for the celery (*Apium graveolens* L.) cell membrane against freezing injury under fungal pathogen stress. However, this benefit is contingent upon the pathogens’ detrimental effects on the permeability of the cell membrane. In addition to membrane stability, in P: SE1 and P: SE2 treated plants; the levels were significantly higher than in non-treated CMV-infected plants by around 78.93% and 88.49%, respectively.

Biotrophic pathogens trigger the SA pathway which results in the transcription of NPR1 and the accumulation of SA signature gene products (PR1, PR2 & PR5), ultimately leading to SAR^81^. Numerous PR proteins have antiviral characteristics^82^. Two different types of PR genes (PR-b1 and PR-2) were tested for their responses to CMV infection across crude extracts SE1 and SE2 treatments in squash plants.

In our study, P: SE1- and P: SE2-treated squash plants (21 DPVI) showed maximum expression of PR-b1 (by 3.09- and 10.37-fold increases, respectively, as compared to the CMV-infected control). While PR-2 was overexpressed by a 1.00-fold increase in CMV-infected control, as compared to mock.

In this situation, tomato plants pre-treated with *Streptomyces pactum* Act12 also exhibited an increase in PR-1 and higher levels of peroxidase (POD) and phenylalanine ammonia lyase (PAL) activities in tomato leaves leading to the formation of SAR against tomato yellow leaf curl virus (TYLCV)^83^.

Applying P: SE2 as a preventive measure before CMV infection showed higher effectiveness in squash plants with PR-b1 overexpression, resulting in about a ten-fold increase compared to the infected group. This indicates that the SA pathway is the primary defense mechanism against CMV in squash plants, supporting findings by Abdelkhalek et al.^42^, who found that the PR-1 gene expression in squash plants treated with rhizobacteria *Paenibacillus polymyxa* strain SZYM culture filtrate was significantly increased by 12- and 6.5-fold in protective and curative treatment groups 21 days after ZYMV inoculation compared to the control group. As well, a novel glycoprotein (GP-1) from *Streptomyces* sp. ZX01 strongly induced pathogenesis-related proteins (PRs) and enhanced systemic resistance against TMV in tobacco plants, but its antiviral mechanism remains unclear^9^.

Some PR proteins, such as PR-1 have been shown to inhibit the replication of certain viruses by interfering with the viral RNA or proteins necessary for replication. PR proteins can limit the spread of viruses within the plants by strengthening cell walls and inhibiting the movement of viral particles between cells.

The PR-2 gene expression was significantly reduced by treatment C: SE1 and C: SE2, with peak values in P: SE1 and P: SE2 (1.07- and 1.66-fold increase, respectively) when compared to the infected control group. PR-2 proteins boost the production of callose which then builds up at plasmodesmata to stop viruses from spreading,

Following P: SE1 and P: SE2 treatment, there was an increase in plant resistance that may have resulted from increased peroxidase activity which is essential for lignification and connecting cell wall proteins, as well as the activation of genes that produce PR proteins which produce phytoalexins and other phenolic compounds^84^. The PR proteins present in uninfected areas of the plant hindered the entry of new viruses and boosted the plant’s ability to resist virus replication and spread^85^. Abdel-Moneim^86^ confirmed that *Streptomyces* strains triggered SAR against viral infections. In accordance to the present results, applying SE1 and SE2 triggers the production of SAR in squash plants to protect them against CMV infection.

Four bioactive compounds with antiviral activities such as 1,3¬Dinitro¬2-imidazolidinone, Nephthoside-1,2’,3’,4’-Tetraacetate, Tetraphenylporphyrinato dichlorotitanium (IV), and L-Lysine detected in ethyl acetate crude extracts (SE1 and SE2) derived from strain ph6 and strain MARH respectively were identified using GC–MS and could prevent virus infection and act as elicitors for SAR against CMV infection in pre-treated squash plants under greenhouse conditions.

The titanium (IV) complexes, including Tetraphenylporphyrinato dichlorotitanium detected in the ethyl acetate extract (SE2) from *Streptomyces* sp. MARH at retention time of 19.48 min (Table 5) has garnered significant interest for its potential biological applications. It has been investigated as an antibacterial agent, biological sensor, tumor cell-killing agent, gene-targeting device, and antibiotic because of its reactive oxygen species-producing capabilities. These reactive oxygen species contribute to the decomposition of bacterial, fungal, algal cells, and viral structure owing to the oxophilic nature of these complexes and their ability to form strong bonds with various biological molecules^87^. These results are in accordance with Chen et al.^7^ who detected that ε-Poly-l-lysine a bioactive compound obtained from *Streptomyces hygroscopicus* had shown significant protective and curative activity against TMV.

Moreover, L-Lysine detected at retention time of 17.01min is an amino acid that primarily interferes with the formation of viral capsid proteins and DNA through competitive antagonism with arginine which is required for certain RNA viruses, as well as through promoting the production of more arginase which increases arginine’s catabolism^88^. Additionally, Ara et al.^89^ discovered that bioactive compounds from *Streptomyces* spp. were successful in reducing local lesions caused by TMV on *Datura metel* leaves.

Imidazolidinones, including 1,3¬Dinitro¬2-imidazolidinone detected in ethyl acetate extract from *Streptomyces variabilis* (SE1) at retention time of 6.37 min (Table 4) are important classes of heterocyclic compounds with strong antiviral properties. These compounds target the capsid protein VP1 of human enterovirus 71 (EV71), preventing viral RNA uncoating. They also impede HCV’s NS3 serine protease^90^. A broad and potent antiviral butanolide [(4S)−4-hydroxy- 10-methyl-11-oxo-dodec-2-en-1, 4-olide] from *Streptomyces* sp. SMU03 was effective against influenza viruses^91^.

These chemical compounds present in the crude extracts (SE1 and SE2) from *Streptomyces variabilis* and *Streptomyces* sp. MARH may be responsible for all their antiviral activity against CMV under greenhouse conditions.

## Conclusions

The results of this study highlight the potential of *Streptomyces* spp. as biocontrol agents against CMV in squash plants and as valuable natural bioactive compounds source. CMV was identified using biological, molecular characterization and sequence analysis. The isolated *Streptomyces variabilis* and *Streptomyces* sp. MARH from the Red Sea in Hurghada, Egypt produced potent secondary metabolites with antiviral properties including 1,3¬Dinitro¬2-imidazolidinone, Nephthoside-1,2’,3’,4’-Tetraacetate, Tetraphenylporphyrinato dichlorotitanium(IV) and L-Lysine. The crude extracts displayed significant inhibition of CMV infection in squash plants through protective treatments. Application of bioactive crude extracts of *Streptomyces* spp. stimulated antioxidant enzymes (APX), (CAT) and the phenolic substances in addition to proline content. Moreover, GC–MS analysis identified bioactive predicted compounds from ethyl acetate extracts of *Streptomyces* spp. which induced PR-b1 and PR-2 genes and enhanced disease resistance against CMV in squash plants. Hence, crude extracts of *Streptomyces variabilis* and *Streptomyces* sp. MARH could be used for the control of CMV. In addition, further exploration of the active metabolites of *Streptomyces* spp. could be conducted in the future and field experiments could also be carried out to promote the application of *Streptomyces* spp. secondary metabolites in the prevention and management of plant viruses.

## Supplementary Information

Supplementary Information.

## Data Availability

Sequence data that support the findings of this study have been deposited in GenBank (https://www.ncbi.nlm.nih.gov/nucleotide/) under the following accession numbers OQ283766, OQ283775 and PX310541.
