# Supplementary material for: Antiviral potential of crude extracts from two Streptomyces spp. against Cucumber Mosaic Virus infection under greenhouse conditions
Source: Sci Rep. 2025 Dec 23;15:44330. doi: 10.1038/s41598-025-31348-9 (PMC12728196; doi:10.1038/s41598-025-31348-9)
Supplement: Supplementary file 1 — Supplementary Information. [file 41598_2025_31348_MOESM1_ESM.pdf]

"Supplementary Material"

Antiviral potential of crude extracts from two *Streptomyces* spp. against *Cucumber Mosaic Virus* infection under greenhouse conditions

Hadeel Osama[1], Mohamed E. El Awady[2], Radwan R. Khalil[1], Amro A. Farrag[3], Ahmed A. Hamed[4] and Mohamed A. Nasr-Eldin[1]*

[1]Department of Botany and Microbiology, Faculty of Science, Benha University, Benha 13511, Egypt

[2]Department of Microbial Biotechnology, National Research Centre, Giza, Egypt

[3]Virus and Phytoplasma Research Department, Plant Pathology Research Institute, Agricultural Research Center (ARC), Giza, Egypt

[4]Microbial Chemistry Department, National Research Centre, Dokki, Cairo, 12622, Egypt

***Corresponding author:** Mohamed A. Nasr-Eldin

E-mail: mohamed.nasreldin@fsc.bu.edu.eg

https://orcid.org/0000-0002-8537-9392

Supplementary Table S1 Different characteristics of high potent activity of *Streptomycete* isolate (ph6)

<table>
<tr><th colspan="8">Morphological and cultural characteristics</th></tr>
<tr><th colspan="2">Spore chain morphology</th><th colspan="2">Spore surface ornamentation</th><th>Color of spore mass</th><th colspan="2">Pigmentation of substrate mycelium</th><th>Diffusible pigment</th></tr>
<tr><td colspan="2">Straight/Flexuous</td><td colspan="2">Smooth</td><td>Gray</td><td colspan="2">Grayish white</td><td>-</td></tr>
<tr><th colspan="8">Physiological and biochemical characteristics</th></tr>
<tr><th colspan="2">Melanin pigment production</th><th colspan="3">Degradation activities</th><th>Nitrate reduction</th><th colspan="2">H_2S production</th></tr>
<tr><th>peptone iron agar</th><th>tyrosine agar</th><th>xanthine</th><th>elastine</th><th>arbutin</th><td rowspan="2">+</td><td colspan="2" rowspan="2">-</td></tr>
<tr><td>-</td><td>-</td><td>+</td><td>-</td><td>-</td></tr>
<tr><th colspan="8">Utilization of sugars</th></tr>
<tr><th>D-fructose</th><th>Sucrose</th><th>Rhamnose</th><th>D-mannitol</th><th>D-xylose</th><th>I-inositol</th><th>Galactose</th><th>L-arabinose</th></tr>
<tr><td>+</td><td>-</td><td>-</td><td>-</td><td>-</td><td>-</td><td>-</td><td>-</td></tr>
</table>

Supplementary Table S2 Different characteristics of high potent activity of *Streptomycete* isolate (MARH)

Morphological and cultural characteristics				
**Spore chain morphology**	**Spore surface ornamentation**	**Color of spore mass**	**Pigmentation of substrate mycelium**	**Diffusible pigment**
Straight/Flexuous	Smooth	White	Grayish white	-

Physiological and biochemical characteristics						
**Melanin pigment production**		**Degradation activities**			**Nitrate reduction**	**H_2S production**
**peptone iron agar**	**tyrosine agar**	**xanthine**	**elastine**	**arbutin**	+	+
+	+	-	-	+		

Utilization of sugars							
**D-fructose**	**Sucrose**	**Rhamnose**	**D-mannitol**	**D-xylose**	**I-inositol**	**Galactose**	**L-arabinose**
-	-	-	+	-	-	+	+

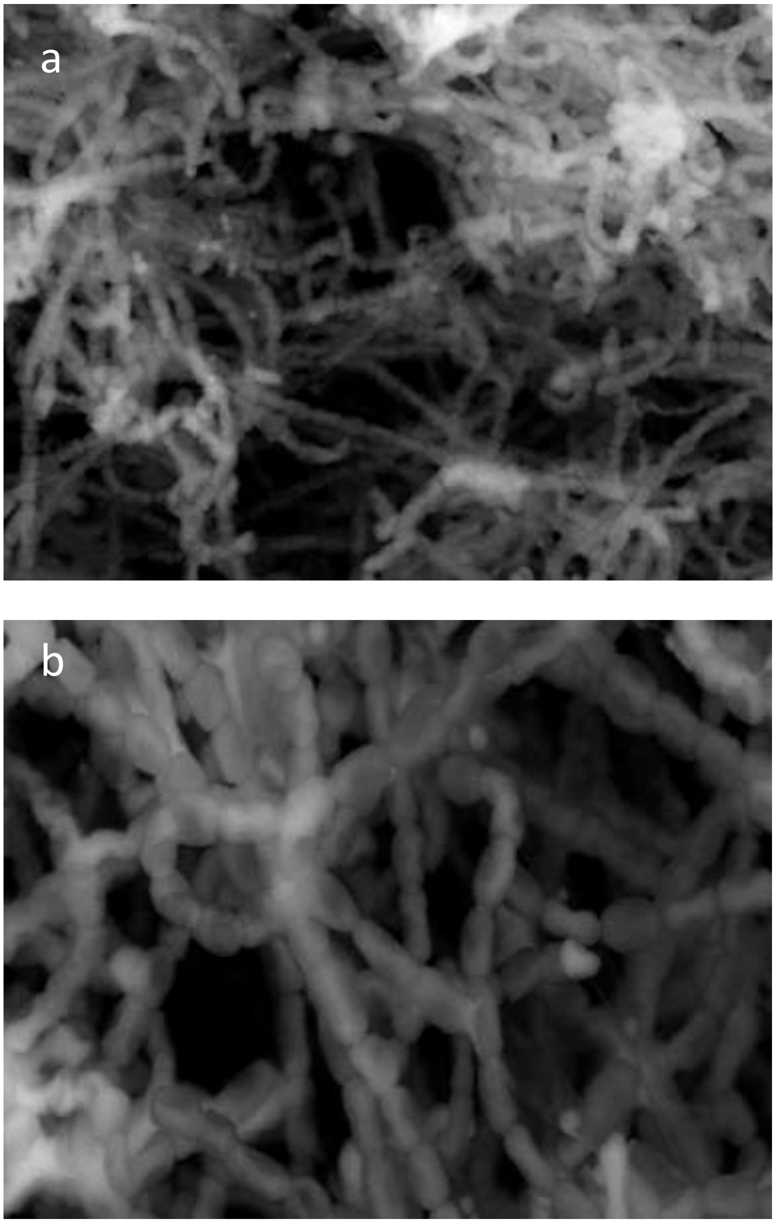

Supplementary Fig. S1 (a) Electron micrograph of *Streptomyces variabilis* strain ph6 and (b) *Streptomyces* sp. strain MARH

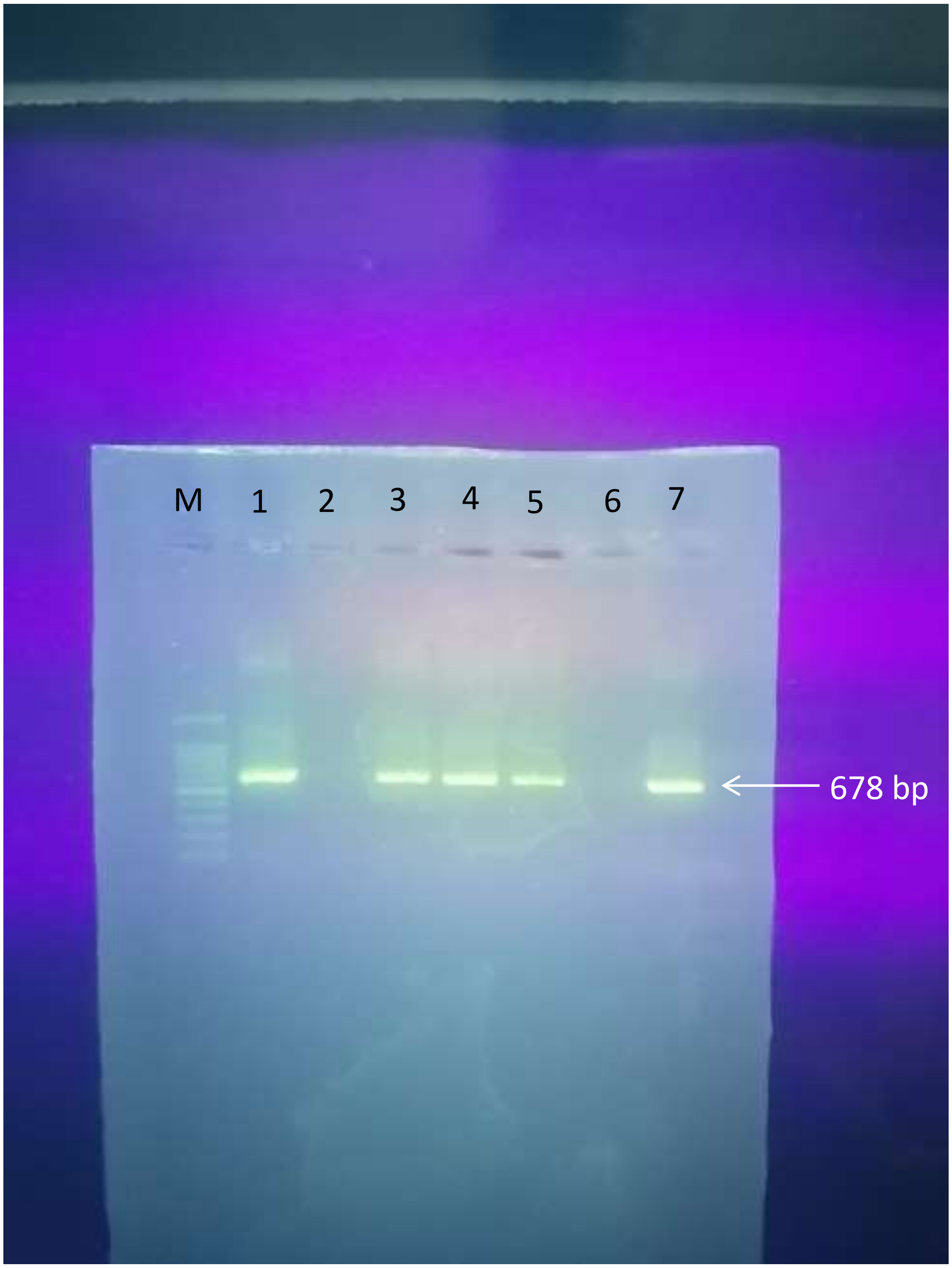

Supplementary Fig. S2 Full-length gel for detection of CMV obtained by RT-PCR with specific primers for the CMV-CP gene detected an amplicon of 678 bp (M, 100bp ladder, 1, 7 positive control, 2, 6 negative control and 3, 4, 5 CMV-infected squash plants).

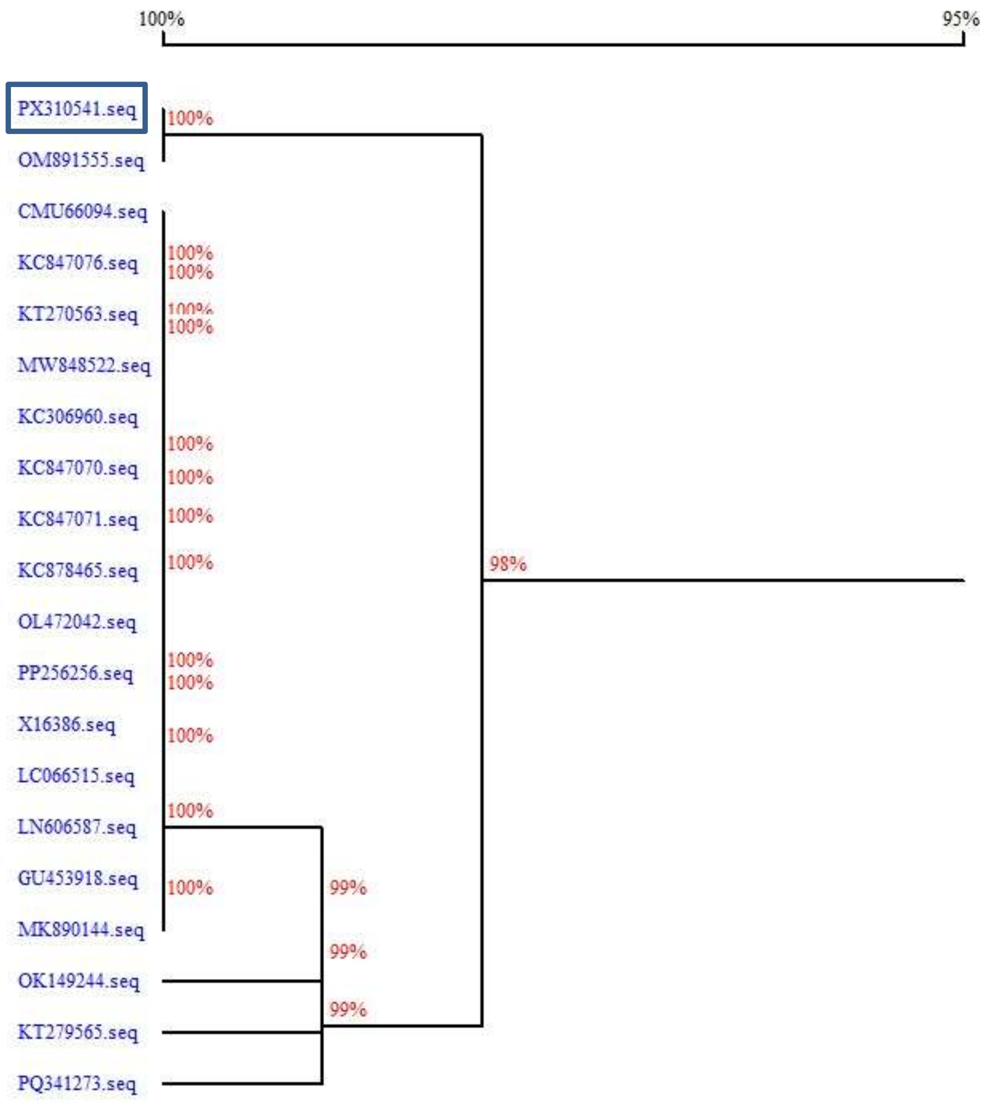

Supplementary Fig. S3 Homology tree derived from the CP nucleotide sequence of CMV with an accession no. of PX310541.

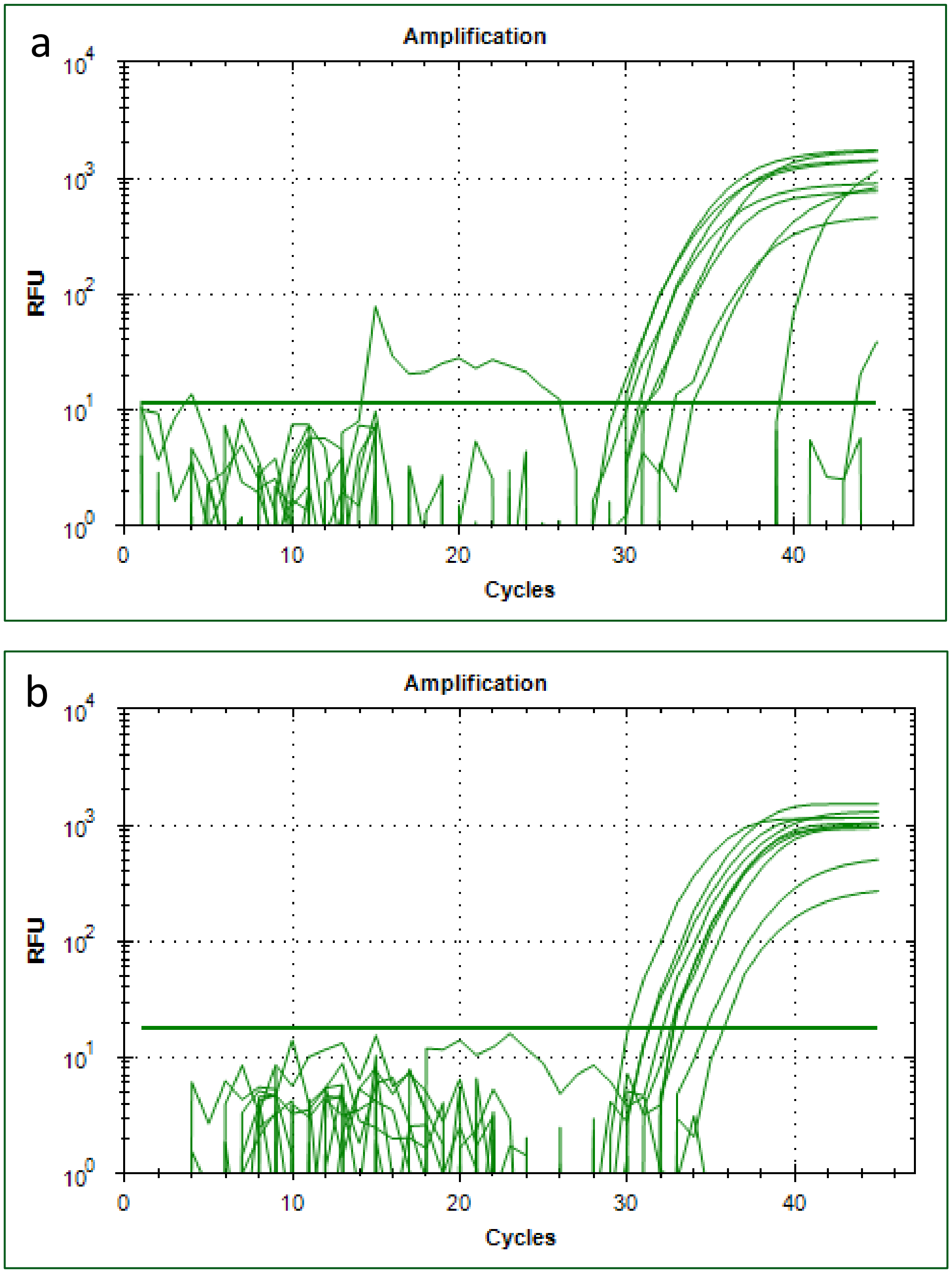

Supplementary Fig. S4 Real-time PCR output, showing different expression of pathogenesis-related (PR-b1) and (PR-2) genes after application of different *Streptomyces* spp. extracts (SE) through curative, protective and inactivation methods at 21 DPVI against CMV infection. Data is the amplification plot detected by BIO-RAD CFX Connect Real-Time PCR Detection System. The PCR was run for 40 cycles. CT value is the PCR cycle number at which the curve intersects the threshold (horizontal line), where fluorescence meets the threshold in the amplification plot. Data were collected from the mean values of Ct of three replicated run by real time PCR and the whole experiment was conducted twice.
